# Cell age dependent concentration of *Escherichia coli* divisome proteins analyzed with ImageJ and ObjectJ

**DOI:** 10.3389/fmicb.2015.00586

**Published:** 2015-06-11

**Authors:** Norbert O. E. Vischer, Jolanda Verheul, Marten Postma, Bart van den Berg van Saparoea, Elisa Galli, Paolo Natale, Kenn Gerdes, Joen Luirink, Waldemar Vollmer, Miguel Vicente, Tanneke den Blaauwen

**Affiliations:** ^1^Bacterial Cell Biology, Swammerdam Institute for Life Sciences, Faculty of Science, University of AmsterdamAmsterdam, Netherlands; ^2^Molecular Cytology, Swammerdam Institute for Life Sciences, Faculty of Sciences, University of AmsterdamAmsterdam, Netherlands; ^3^Department of Molecular Microbiology, Institute of Molecular Cell Biology, VU UniversityAmsterdam, Netherlands; ^4^Centre for Bacterial Cell Biology, Institute for Cell and Molecular Biosciences, Newcastle UniversityNewcastle upon Tyne, UK; ^5^Centro Nacional de Biotecnología-Consejo Superior de Investigaciones CientíficasMadrid, Spain; ^6^Department of Biology, University of CopenhagenCopenhagen, Denmark

**Keywords:** non-destructive marking, divisome, image analysis, immunolocalization, FtsZ, PBP1B, LpoA, FtsN

## Abstract

The rod-shaped Gram-negative bacterium *Escherichia coli* multiplies by elongation followed by binary fission. Longitudinal growth of the cell envelope and synthesis of the new poles are organized by two protein complexes called elongasome and divisome, respectively. We have analyzed the spatio-temporal localization patterns of many of these morphogenetic proteins by immunolabeling the wild type strain MC4100 grown to steady state in minimal glucose medium at 28°C. This allowed the direct comparison of morphogenetic protein localization patterns as a function of cell age as imaged by phase contrast and fluorescence wide field microscopy. Under steady state conditions the age distribution of the cells is constant and is directly correlated to cell length. To quantify cell size and protein localization parameters in 1000s of labeled cells, we developed ‘Coli-Inspector,’ which is a project running under ImageJ with the plugin ‘ObjectJ.’ ObjectJ organizes image-analysis tasks using an integrated approach with the flexibility to produce different output formats from existing markers such as intensity data and geometrical parameters. ObjectJ supports the combination of automatic and interactive methods giving the user complete control over the method of image analysis and data collection, with visual inspection tools for quick elimination of artifacts. Coli-inspector was used to sort the cells according to division cycle cell age and to analyze the spatio-temporal localization pattern of each protein. A unique dataset has been created on the concentration and position of the proteins during the cell cycle. We show for the first time that a subset of morphogenetic proteins have a constant cellular concentration during the cell division cycle whereas another set exhibits a cell division cycle dependent concentration variation. Using the number of proteins present at midcell, the stoichiometry of the divisome is discussed.

## Introduction

*Escherichia coli* is a Gram-negative rod shaped bacterium that divides by binary fission. The new daughter cells will first elongate in length before a new division cycle is initiated at a cell age dependent on cell mass (Taheri-Araghi et al., 2015). Consequently, fast growing cells that are much longer than slowly growing cells initiate division almost immediately after birth. Large protein complexes that are termed elongasome and divisome synthesize and hydrolyze peptidoglycan during cell elongation and cell division, respectively (Egan and Vollmer, 2013; van der Ploeg et al., 2013). These protein complexes share some of their proteins (Mohammadi et al., 2007; White et al., 2010; van der Ploeg et al., 2013), and many of the proteins have their own enzymatic activities, which categorize the elongasome and divisome as hyperstructures (Norris et al., 2007). These hyperstructures are not assembled and then kept stable like the ribosomes, they are rather dynamic and can associate cell cycle dependent. It is therefore relevant for the understanding of the organization of both processes to determine their composition and cellular localization as a function of the bacterial cell division cycle age (cell age).

### Observing Cells in Steady-State Growth

*Escherichia coli* grows exponentially making it possible to access cell age dependent information without the need for synchronizing the cells. In liquid medium growing cells that are repeatedly diluted in pre-warmed medium at an early exponential phase will develop a constant metabolism (Dennis and Bremer, 1974). From then on, the number of cells in the culture will increase just as fast as the total mass or optical density of the cells in the culture. As a result, both the average mass of the cells in the culture and their age frequency distribution, are constant, the hallmarks of steady state growth. Because the *E. coli* cell diameter is constant, it is possible to determine the age of an individual cell by its length. High quality phase contrast imaging in combination with image analysis allows the conversion of a length distribution to an age distribution of large numbers of cells comprising all ages. Precise spatio-temporal information on bacterial proteins during the cell cycle can be obtained using specific antibodies conjugated to fluorophores.

### Coli-Inspector

A specialized software project (Coli-Inspector) was developed for the analysis of the morphometrical and fluorescence related properties of the immunolabeled proteins. Measurements included cell length, cell diameter, constriction sites, and spatial distribution of fluorescence along the cell axis. This information is extracted from sets of phase contrast and fluorescence images that are organized as hyperstacks. In order to acquire and manage this multitude of parameters across many images in an integrated way, we used ImageJ (Schneider et al., 2012) in combination with the ObjectJ plug-in.

ObjectJ focuses on the organization of image-analysis tasks using an integrated approach. Central to a task is a project file that dynamically links all related components together: a user-defined palette for non-destructive markers, color-coded hierarchical vector objects across many images that are linked to the project, qualifiers for creating subsets of results, and the macros that are in use. The project stores all previous analysis results and at any time the user has the flexibility to extract different sets of results from marked locations such as intensities and spatial parameters.

An important feature ensures that every step during the analysis is clearly visualized with the possibility to intercept or override automatic methods, which helps to eliminate artifacts at an early stage.

ObjectJ helps to keep the desktop clean by integrating all relevant information in the project file instead of creating additional files. In most cases, graphs and numerical output can be displayed transiently from the newest data set without the need to send files to an external (spread sheet) program, which means that the interconnection of cell data and their link to the images remains intact.

**FIGURE 1:**
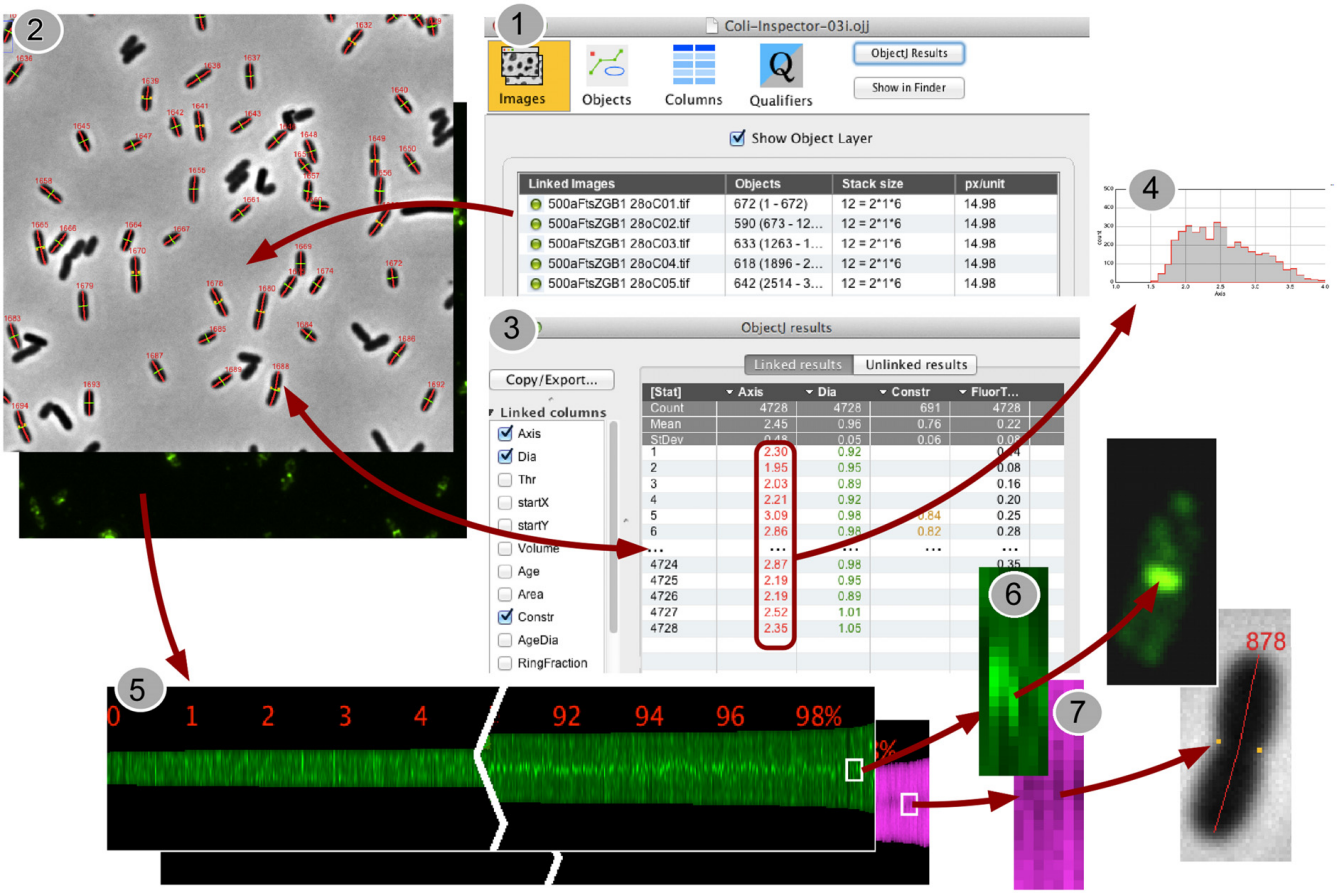
**Back-and-forth navigation between individual image processing steps. (1)** In the project window “Coli-Inspector-03i.ojj,” the panel for “Linked Images” is active and shows the double-clickable names of the images that are linked and thus intended to be analyzed. **(2)** Via embedded macro commands, the cells in all linked images can be marked as “composite objects,” which consist of different markers for cell axes (red), cell diameters (green), and constriction sites (yellow dots). Markers are displayed transiently upon the images, while being stored and managed in the project file. **(3)** Calculated cell properties are displayed in the “ObjectJ Results Table” that is part of the project file. Each cell occupies one row. Any number of columns can be defined to store the desired morphometric or intensity related results. Result rows are bi-directionally linked to the corresponding cells for fast back-and-forth navigation. **(4)** ObjectJ result columns include column statistics and can be directly visualized as histograms. **(5)** A Map of Profiles is created that visualizes all cell profiles along their axes as 1-pixel-wide columns in a floating-point hyperstack. The height of a column corresponds to the cell length in pixels, and each pixel holds the local fluorescence (green) or the local diameter (magenta). Here, the profile map is sorted from short to long cells and, as steady state population, exponentially calibrated in age from 0 to 100%. Thus it visualizes the change of fluorescence distribution along the cell during cell cycle time (green), and the development of the constriction before the cell divides (magenta band becomes darker due to smaller diameter at the constriction site). **(6,7)** Clicking in the Map will expose the corresponding cell, which allows quickly locating artifacts or special phenomena.

A special feature of Coli-Inspector is the creation of profile maps (**Figure 1**, Panel 5), which visualizes the spatio-temporal distribution and correlation of different fluorophores along the cell axis. Optionally, the profile map can arrange profiles for each cell in a way that the pole with the stronger fluorescence in the “leader channel” always points to the same side (**Figure 3**). Fluorescence in any other channel (“follower channel”) is not correlated if the collective distribution along the cell axis remains symmetrical due to the random orientation during acquisition. This is illustrated by the immunolabeled Min system that prevents polar divisions.

### Protein Localization Analysis

The Coli-Inspector project was tailored for spatio-temporal protein localization analysis and is, like ImageJ and ObjectJ, free and open source^1^. A user manual is available online.

Using the Coli-Inspector project, we determined the localization of PBP1B, PBP1A, PBP3, PBP5, LpoB, LpoA, FtsB, FtsK, FtsN, FtsZ, ZapA, ZapB, ZipA, MinC, and MinD as a function of cell age in the wild type strain MC4100 grown in minimal glucose medium at 28°C. All data are directly comparable because the cells were grown to the same steady state. Since every medium and growth temperature results in a different steady state, the timing of protein localization cannot be extrapolated to other growth rates and conditions. However, the general organization of morphogenesis is probably similar under at least a variety of laboratory conditions.

Using the steady state cell growth approach, we have previously shown that the maturation of the divisome occurs in two clearly separated steps (den Blaauwen et al., 1999; Aarsman et al., 2005; van der Ploeg et al., 2013). In the first step, the proto-ring is assembled at midcell. The tubulin homologue FtsZ polymerizes in a ring-like structure underneath the cytoplasmic membrane at midcell. FtsA and ZipA localize simultaneously with the Z-ring (Rueda et al., 2003) and tether the Z-ring to the cytoplasmic membrane. Other proteins such as ZapA help to organize the ring during its status nascendi (Mohammadi et al., 2009; Bisicchia et al., 2013a). ZapB binds to itself, to ZapA and to FtsZ and seems to function as a sensor between the terminal region of the chromosome and the Z-ring (Espeli et al., 2012). In the second step with some time delay (Aarsman et al., 2005), all other cell division proteins are recruited, including FtsK, PBP3, PBP1B, FtsB, and FtsN. The role of these proteins will be further discussed in the result section.

The combination of steady state growth, the fluorescent immunolabeling of endogenous proteins, and the unique features of Coli-Inspector were used in this study to assess the cellular protein concentration of the above-mentioned morphogenetic proteins as a function of the bacterial cell age. Many proteins are present at a constant cellular, not necessarily uniformly localized, concentration. Interestingly, several proteins other than FtsZ [whose transcription is known to be regulated (Garrido et al., 1993)] appear to have a varying concentration.

The recent publication of Li et al. (2014) reports the number of molecules of each protein synthesized in one generation in *E. coli* as measured by ribosome profiling. These data allowed us to convert fluorescence arbitrary units into the number of protein molecules and to determine the number of proteins at midcell for each immunolabeled divisome protein. The resulting data were used to discuss the stoichiometry of the cell envelope synthetic machinery during the constriction process.

## Materials and Methods

### Growth Conditions and Media

*Escherichia coli* K12 cells were grown to steady state in glucose minimal medium (Gb1) containing 6.33 g of K_2_HPO_4_.3H_2_O, 2.95 g of KH_2_PO_4_, 1.05 g of (NH_4_)_2_SO_4_, 0.10 g of MgSO_4_.7H_2_O, 0.28 mg of FeSO_4_.7H_2_O, 7.1 mg of Ca(NO_3_)_2_.4H_2_O, 4 mg of thiamine, 4 g of glucose and 50 μg of required amino acids per liter pH 7.0 at 28°C. MC4100 (LMC500) requires Lys for growth in minimal medium. Absorbance was measured at 450 nm with a 300-T-1 spectrophotometer (Gilford Instrument Laboratories Inc.). Steady state growth was achieved by dilution of an over night culture 1:1000 in fresh prewarmed medium of 28°C. The cells were allowed to grow up to a density of 0.2 and then diluted again in prewarmed medium. This procedure was repeated during 40 generations of exponential growth. The mass doubling time of MC4100 is 80 min under these conditions. The overnight dilution was calculated using the equation: $D=2^{\mathrm{t/Td}}(\frac{{\mathrm{OD}}_{\mathrm{now}}}{{\mathrm{OD}}_{\mathrm{des}}})$, where D is the required dilution of the culture to obtain the desired optical density (OD_des_) after t minutes, and Td is the mass doubling time in min. OD_now_ is the optical density of the culture to be diluted. The steady state cultures were fixed by addition of a mixture of formaldehyde (f.c. 2.8%) and glutaraldehyde (f.c. 0.04%) to the shaking water bath. This gives an osmotic shock that does not affect the localization of membrane or cytosolic proteins (Hocking et al., 2012; van der Ploeg et al., 2013). Unfortunately, periplasmic proteins that are freely diffusing are shocked toward the poles. Therefore, the procedure is not suitable for immunolabeling of periplasmic proteins and if used, their localization pattern should be verified using fluorescent protein (FP) fusions and live imaging.

### Immunolabeling

Immunolabeling of the cells was performed as described (Buddelmeijer et al., 2013). Antisera were either pre-purified using cells of a deletion strain (**Table 1**) of the particular protein against which the antiserum was directed, or the specific IgG was purified using the native protein against which it was directed (Karczmarek et al., 2007; Typas et al., 2010). In brief, formaldehyde/glutaraldehyde fixed and Tx100/lysozyme permeabilized cells were incubated for 1 h at 37°C with purified polyclonal antibodies directed against FtsK, FtsN, FtsB, FtsZ, ZipA, MinC, MinD, PBP3, PBP5, PBP1B, PBP1A, LpoB, LpoA, ZapA, all diluted in blocking buffer. ZapB was immunolabeled with Fabs conjugated to Cy3. As secondary antibody, donkey anti-rabbit conjugated to Cy3 (Jackson Immunochemistry, USA) diluted 1:300 in blocking buffer (0.5% (wt/vol) blocking reagents (Boehringer, Mannheim, Germany) in PBS) was used, and the samples were incubated for 30 min at 37°C. For immunolocalization, cells were immobilized on 1% agarose in water slabs coated object glasses as described (Koppelman et al., 2004) and photographed with a Coolsnap *fx* (Photometrics) CCD camera mounted on an Olympus BX-60 fluorescence microscope through a 100x/N.A. 1.35 oil objective. Images were taken using the program ImageJ with MicroManager^2^.

**Table 1:** Used strains and their genotypes.

Strain name	Characteristics	Genotype	Source
MC4100	Wild type	F^-^, *araD139,* Δ*(argF-lac)U169deoC1, flbB5301, ptsF25, rbsR, relA1, rpslL150, lysA1*	Taschner et al. (1988)
BW25113	Wild type	F^-,^Δ*(araD-araB)567,* Δ*lacZ4787*(::rrnB-3), λ^-^, *rph-1,* Δ*(rhaD-rhaB)568, hsdR514*	Baba et al. (2006)
PA340-678	ΔMreBCD	F^-^, *argH1, thr-1, leuB6, ghd-1, gltB31, thi-1, lacY1, gal-6, xyl-7, ara-14, mtl-2, malA1, rpsL9, tonA2*	Wachi et al. (1987)
CS12-7	ΔPBP5	W1485 *rpoS rpH dacA::kan* 512-1	Potluri et al. (2010)
LMC1084	ΔMinCDE	PB114 Δ*minB*::Km(R), *dad*R1, *trpE61, trpA62, tna5, purB,L-*^+^	de Boer et al. (1989)
BW25113 ΔlpoA	ΔLpoA	BW25113 Δ*lpoA*	Baba et al. (2006)
BW25113 ΔlpoB	ΔLpoB	BW25113 Δ*lpoB*	Baba et al. (2006)
JW3359 mrcA	ΔPBP1A	BW25113 Δ*mrcA*	Baba et al. (2006)
JW0145 mrcB	ΔPBP1B	BW25113 ΔmrcB	Baba et al. (2006)
LMC3143	ΔZapA	LMC500 Δ*zapA*	Mohammadi et al. (2009)
MC1000 ΔZapB	ΔZapB	Δ*zapB* Δ (*ara-leu*) Δ*lac rpsL*150	Ebersbach et al. (2008)
CH5/pCH32	ZipA depletion	PB103 *zipA::aph*/*aadA*^+^ *repA*(Ts) *ftsZ*^+^*zipA*^+^ *recA::Tn10*	Hale and de Boer (1999)

### Image Analysis

Phase contrast and fluorescence images were combined into hyperstacks using ImageJ^3^ and these were linked to the project file of Coli-Inspector running in combination with the plugin ObjectJ^4^. The images were scaled to 14.98 pixel per μm. The fluorescence background has been subtracted using the modal values from the fluorescence images before analysis. Major analysis steps are given in the Section “Results,” and the full Coli-Inspector documentation can be found at https://sils.fnwi.uva.nl/bcb/objectj/examples. Slight misalignment of fluorescence with respect to the cell contours as found in phase contrast was corrected using Fast-Fourier techniques. The fluorescence image was translated in x–y direction so that fluorescence measured under all cell contours reached a maximum^5^.

### Data Analysis

Cells are assumed to have rotational symmetry, where the mid-line as detected from the cell contour in phase contrast represents the cell axis. Partial or entire cell volume is obtained by the integration of 1-pixel-thick disks with local diameter along the cell axis. Envelope area is obtained by contour rotation. Fluorescence values are derived from the second channel of the profile map, where each cell is represented as a vector (1-pixel wide column). Each pixel contains the entire fluorescence of a 1-pixel-thick disk including light detected slightly outside the contour due to the point-spread function. The sum of all vector elements (pixels) is displayed as FluorTotal. The concentration of the fluorescence per cell (ConcTotal) or the concentration in the envelope (ConcWall) was calculated by dividing the FluorTotal by either the cell volume (for FtsZ, ZapA, and ZapB), or by the envelope area for all other proteins that are cytoplasmic membrane bound or inserted. In order to relate fluorescent light quantities to absolute numbers of protein molecules, the conversion factor *F* was calculated by dividing the integrated fluorescence by the number of proteins of the average cell. The number of involved protein molecules could then be calculated for an individual cell or even a part of it. Midcell was defined as the central part of the cell comprising 0.8 μm of the axis. From either cell part, midcell and remaining cell, the volume, the integrated fluorescence, and thus the concentration of fluorophores can be calculated. The difference of the two concentrations is multiplied with the volume of midcell. It yields FCPlus (surplus of fluorescence) and, via factor *F*, MolsFCPlus (surplus of protein molecules at the cell center). These values are positive or negative for higher or lower concentrations in the center, respectively. For age calculation, all cell lengths are sorted in ascending order. Then the equation

$$age=ln(1-0.5^{*}rank/(nCells-1))/ln(0.5)$$

is used, where *rank* is a cell’s index in the sorted array, *nCells* is the total amount of cells, and *age* is the cell’s age expressed in the range 0.. 1. For explanation of the most important parameters used in this study see **Table 2**.

**Table 2:** Overview of the most important parameters used for the analysis of the spatio-temporal localization of the immunolabeled proteins.

Parameter	Description	Unit
Age	Cell age based on cell length	(0–100) %
*F*	Conversion factor	proteins/FluorUnit
Fluortotal	Integrated fluorescence of cell	FluorUnit
Volume	Total cell volume (sum of disk volumes)	μm^3^
ConcTotal	Concentration of fluorescent material in cell volume	FluorUnit/μm^3^
CellWall	Area of cell envelope	μm^2^
Area	Area of cell projection (contour as obtained from phase contrast image)	μm^2^
ConcWall	Concentration of fluorescent material in cell envelope	FluorUnits/μm^2^
MidCell Volume	Cell compartment ± 0.4 μm from cell center	μm^3^
FCPlus	Surplus of fluorescence in cell center compared to the rest of the cell	FluorUnit
MolsCPlus	Molecules in Center surplus gives the number of molecules in the cell center that are in surplus compared to the rest of the cell (calculated from FCPlus ^∗^ *F*)	molecules

## Results and Discussion

### Coli-Inspector for Multi-Parameter Image Analysis

The settings and macro commands of the Coli-Inspector project, in combination with the ObjectJ plugin, made it possible to perform a large number of different experiments, each based on multi-parameter measurements of 1000s of cells. The “Qualifying” mechanism allowed addressing subsets of cells that then could be used for selective browsing, creating plots, or identifying artifacts. ObjectJ manages the interconnection of individual data structures via the “project file” without creating auxiliary files, which keeps the desktop clean. Only the project file (“.ojj” extension), together with the hyperstacks to be analyzed (linked images), need to be in the same directory. It was not necessary to rely on an external spreadsheet program, which would have disconnected the results from the marked cells in the images. Coli-Inspector’s specific macro commands appear in the ObjectJ menu in approximately the order they are typically invoked.

When the project file is opened in ImageJ, four different panels appear and can be selected via icons: “Images,” “Objects,” “Columns,” and “Qualifiers” (**Figure 1**, Panel 1). The image files must then be “linked” to the project, e.g., by dragging them from the project folder onto the “Images” icon or its panel. They must be 3D or 4D hyperstacks, where the third dimension contains “channels” for phase-contrast and fluorescence images (**Figure 1**, Panel 2). In the fourth dimension, different field views can be stored as “frames.” Scaling is required in pixels per micrometer. This information is reflected in the “Images” panel under “stack size” and “px/unit,” respectively. The images in the downloadable example project conform to these requirements.

### Analysis of Cells

Typically, the user starts with the command “Mark Filaments” and checks whether rejections (e.g., due to clustered cells) in the first few images are plausible, and will then continue with full automatic analysis of all remaining images at a speed of ∼500 cells per minute. Cells are analyzed by first calling ImageJ’s particle analyzer and then by performing additional shape recognition. A perpendicular slit-shaped window is moved from the cell’s center toward either end for detecting the possibly curved cell axis. Then a number of shape parameters are tested for accepting or rejecting the cell. In case of rejection, a temporary yellow text overlay above the cell displays the conflicting criterion, so the user can visually verify the efficiency of the current shape criteria.

In contrast to ImageJ, that does not support composite regions of interest, ObjectJ can handle hierarchical non-destructive objects (for marking cells) and can either address the entire object or subordinate parts of it (“items”) for further analysis. If a cell is accepted, it is treated as a single object and is marked with a segmented line item of type “Axis” (cell length in red), and a line item of type “Dia” (mean diameter in green, **Figure 1**, Panel 2). The corresponding numerical data will automatically appear in one row per cell in the ObjectJ “results table” (**Figure 1**, Panel 3). More items such as constriction markers can optionally be added later. Rather than using ImageJ’s built-in overlay technique, which linearly stores the ROI information in the image file or in the ROI manager and which is optimized for single images, ObjectJ manages all information centrally by the project file. Populations that extend across many hyperstacks can be marked without putting any organizational burden upon the user. Manual detection of artifacts that are left over from imperfect shape recognition takes into account that they often appear at either end of the spectrum of property values (e.g., very thick or very thin cells). With a single keystroke any subpopulation could be browsed in the order of any sorted parameter such as length, diameter or derived result, refining the power of automatic classification with rapid visual inspection. Deletion of undesired objects can also be performed with a keystroke, removing all markers of the selected cell. The ObjectJ “results table” is then automatically updated and allows the observation of statistics or the creation of histograms via the contextual menu connected to each column title (**Figure 1**, Panel 4).

### Map of Profiles

Additional information of the cell population is stored in the “Map of Profiles,” which is a 32-bit (floating point) stack holding as many channels as the acquired images, and which is stored in the “project folder.” For each cell, one slot is arranged containing a vertically centered pixel column whose height corresponds to the cell length. In case of fluorescence, a pixel in the Map contains the integrated brightness of a 1-pixel-thick disk at the corresponding axis position (**Figure 1**, Panel 6). In case of phase contrast, a pixel contains the local cell diameter. For example, the smaller diameter at a cell constriction site will be translated into less brightness and appear dark in the center (**Figure 1**, Panel 7).

Creating Sorted and Qualified Maps was useful to show the cells in growing order from left to right. As length is related to age, the development pattern during the cell cycle can be observed, such as the creation of the Z-ring (**Figure 1**, Panel 5) or the constriction process toward the end of the cell cycle.

### Collective Profile

A collective profile is created from all cell profiles in a Map. They are first resampled to a normalized cell length of 100 data points, and then averaged to a single plot. Optionally, the collective profiles of several channels can be displayed in the same graph. In case of a steady state population, the cell cycle time can be resolved in a number of age groups. For example, specifying 10 age groups will create a stack of 10 profiles that shows development stages during a typical cell cycle.

### Integrated Data Analysis

The Map of Profiles is a useful intermediate data set. It can visualize the longitudinal fluorophore distribution depending on age, and it allows deriving results and plots that describe individual age groups. In general, due to the integrated concept of ObjectJ and its “Qualifying” feature, histograms and scatterplots of any subpopulation can be created inside ImageJ while keeping the link to the images intact.

**FIGURE 2:**
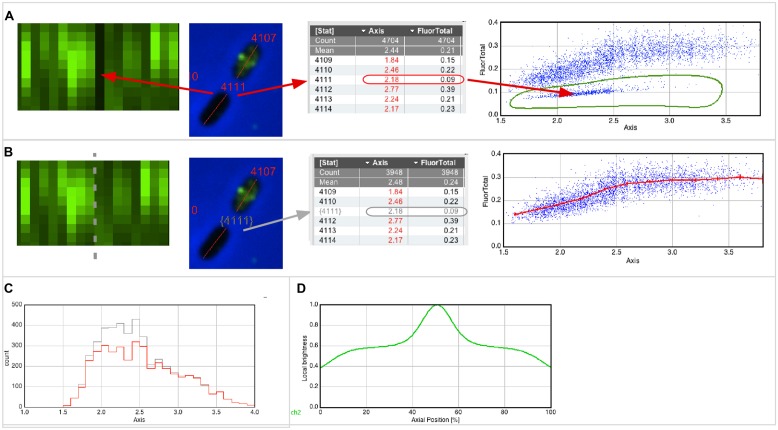
**Qualifying and plotting. (A)** The Map of fluorescence (left) shows a vertical black line, indicating low fluorescence (FluorTotal = 0.09) for cell #4111 that obviously did not permeabilize properly. In the scatter plot (right), which shows fluorescence versus axis length, similar cells appear as a cloud of dots in the lower part. Using a hand-drawn region of interest (ROI), these cells can be easily enclosed and selected. **(B)** The software allows temporarily disqualifying those cells whose dots are in the hand-drawn ROI. Disqualified cells will appear with a gray number label both in their images and in the results table, and they do not contribute to statistics, plotting and sorting. The Map of fluorescence (left) can be updated to show qualified cells only, and can be sorted for axis length. The scatter plot (right) is redrawn to include qualified cells only, and shows a line through markers of the mean value of 0.25 μm length bin size with error bars of 95% confidence. **(C)** Distribution of cell lengths of all and qualified-only cells in gray and red, respectively. **(D)** Collective normalized profile of qualified cells, showing the fluorescence distribution versus the relative position along the cell axis.

For example, **Figure 2A** (left) shows that cell #4111 appears as a black slot in the Map, indicating very low fluorescence. The image of this cell, as well as its numerical properties, can be displayed with a single click (red arrows). When creating a scatter plot “Fluorescence vs. Axis,” similar non-fluorescent cells appear as a separate cloud of points, which can be selected with a hand-drawn region of interest (ROI; **Figure 2A**, right). The low fluorescence could be explained with low inflow of fluorophores due to weak permeabilization (however, for experiments described here below, only cultures in which all cells were permeabilized were used). **Figure 2B** shows how these cells could be excluded from data analysis. Disqualifying cells uses gray color for labels and results (gray arrows) and excludes them from the statistics, plots (**Figure 2B**, right; **Figures 2C,D**) and optionally from the Map (**Figure 2B**, left). As long as non-qualified cells are not deleted from the results, the total population will appear in gray and the qualified population will appear in red in any histogram created from the result table (**Figure 2C**).

### Asymmetrical Localization

**FIGURE 3:**
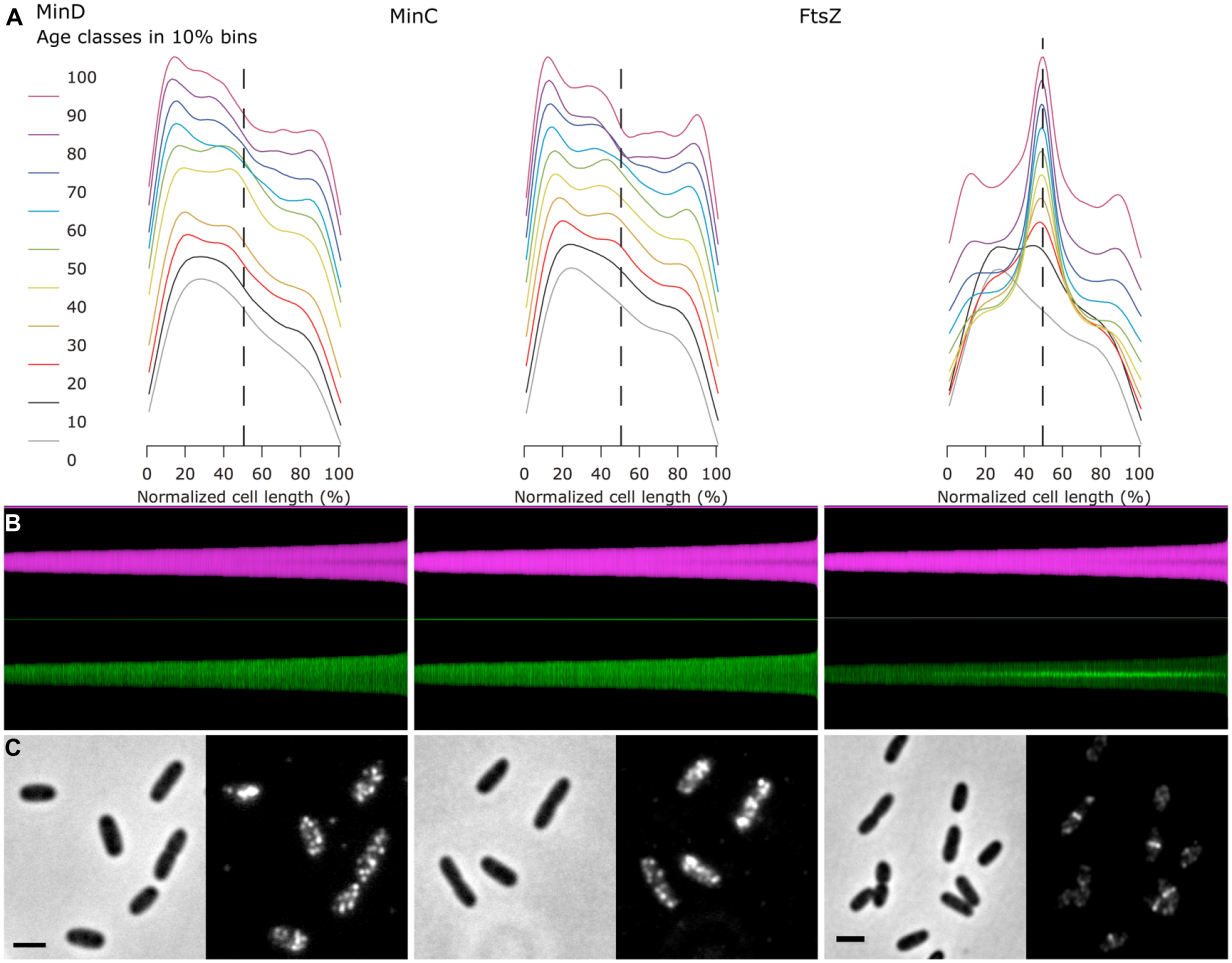
**Fluorescence profiles of immunolabeled endogenous MinC, MinD, and FtsZ show cell division cycle dependent localization. (A)** For each of the three proteins, the fluorescence profiles along the cell axis are shown for 10 age classes. The profiles are asymmetric, as the brighter pole was oriented toward left before averaging. Plots are vertically stacked with an increment of 0.1 for better visualization. Dashed line indicates cell center. **(B)** Map of diameter profiles (magenta) and fluorescence profiles (green, bright pole upward). Cells are sorted for length, ascending from left to right. **(C)** Channel pairs of phase contrast (left) and fluorescence (immunolabeled proteins, right) are shown. The scale bar equals 2 μm.

A “collective profile” appears symmetrical due to the random up/down orientation of the cells in the Map (**Figure 2D**). However, cells that show an asymmetrical distribution of fluorescence along the cell axis, i.e., having a bright and a dark pole, are valuable candidates to further study the spatial correlation of different fluorophores. Therefore, a command is available to orient the cells in the map so that the bright pole in a chosen “leader channel” always points upward (**Figure 3B**). The asymmetry of that channel is thus preserved after averaging, which results in a collective profile with its median in the left half (**Figure 3A**). Any asymmetry in the profile of a “follower channel” indicates that fluorophore localization is correlated with the leader channel, whereas symmetry suggests an independent process. Collective profiles can also be created from individual age classes to resolve the fluorophore localization during the cell cycle (**Figure 3A**).

### The Min System

As an example of the use of the asymmetrical profiles, the MinD and MinC proteins of the Min system of *E. coli* were immunolabeled. The Min system consists of three proteins (see for a review Lutkenhaus et al., 2012): first, the ATPase MinD that binds to the cytoplasmic membrane of the cell poles with an amphipathic helix when it is in the ATP bound form; second, the FtsZ polymerization inhibitor MinC that is recruited by MinD to the membrane; and third, MinE, which stimulates the ATPase activity of MinD. This stimulation of the ATPase activity by MinE causes the release of MinD and MinC from the membrane. Because the majority of the Min proteins are localized at one pole, the release of MinCD causes the proteins to move to the opposite cell pole, where they attach again to the inner membrane. The subsequent stimulation of the ATPase activity of MinD by MinE at this pole causes the cycle to start again, resulting in a regular oscillation of the three proteins from one pole to the other. As a result, FtsZ polymerization is inhibited near the cell poles. This oscillation behavior has been demonstrated *in vivo* using Min FPs fusions (Raskin and de Boer, 1997, 1999a,b) and also *in vitro* using the isolated Min proteins (Loose et al., 2011; Arumugam et al., 2014). Because of the oscillation, all Min proteins could theoretically end up in one of the daughter cells during division. However, *in vivo* studies using FP fusions to MinD (Juarez and Margolin, 2010), MinC and MinE (Ventura and Sourjik, 2011) have shown that the Min proteins become equally distributed between the new born daughter cells, because the oscillation wave is split in two before the closure of the septum. Assuming that the MinC and MinD proteins would on average be present at higher concentrations in the cell poles, we used this characteristic to demonstrate the use of Coli-Inspector’s ability to sort cells according to age, together with the analysis of asymmetric fluorescence profiles.

For this purpose, wild type cells grown to steady state in minimal glucose medium at 28°C were labeled with anti-MinD, -MinC, and -FtsZ (**Figure 3**). Subsequently, the cells were measured and sorted according to cell length with fluorescent profiles, in which the brighter pole is always pointing upward (**Figure 3B**). In an average map of fluorescence profiles with random orientation of the brighter pole, the polar localization of the Min proteins would not be very obvious (**Figure 3C**), but after orienting the brighter poles pointing upward and plotting of the profiles in 10% age classes, the similar asymmetric polar localization of MinC and MinD becomes obvious (**Figure 3A**).

In conclusion, the Coli-Inspector features enable the comparison and verification of the localization behavior of the endogenous Min proteins with that of the FP-Min protein fusions. In addition, information about the age dependent localization pattern could be obtained.

### Concentration of Cell Division Proteins During the Division Cycle

**FIGURE 4:**
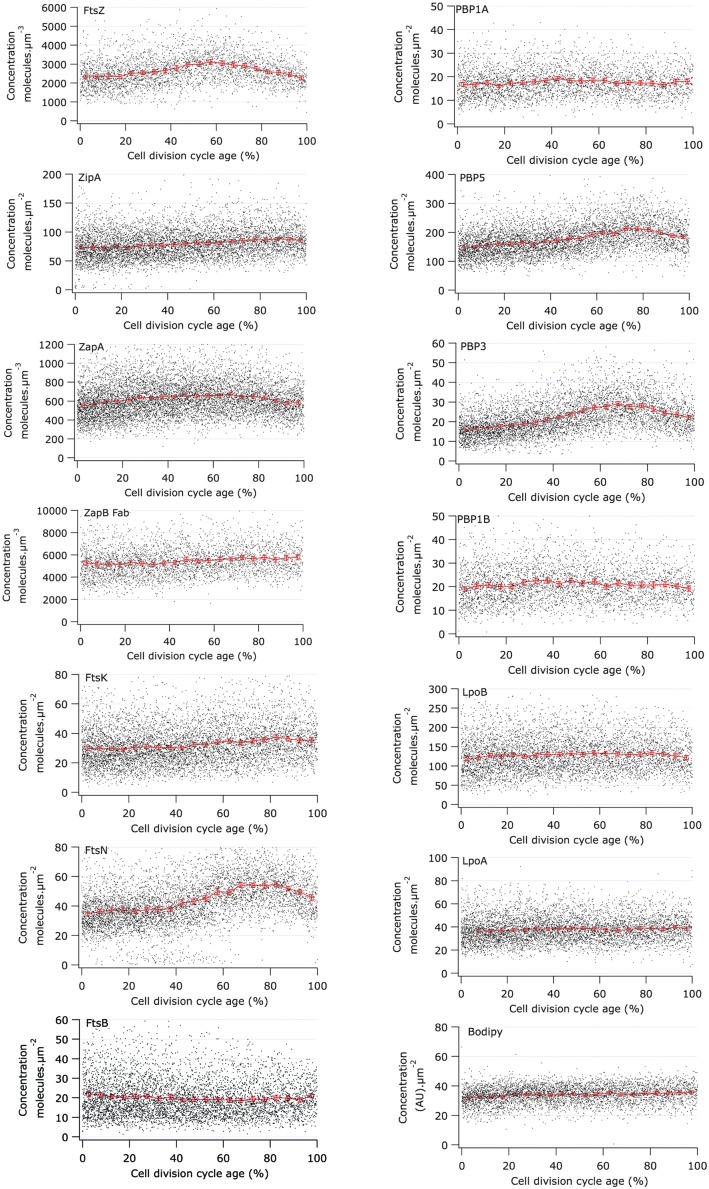
**Concentration of morphogenetic proteins as function of the bacterial cell division cycle.** For each graph the concentration of the indicated protein is plotted against the cell age in %. The black dots are the data for each individual cell. The red line and markers are the mean value of 5% age bins and the error bars indicate the border of the 95% confidence interval. The concentration of FtsZ, ZapA, and ZapB is plotted per volume unit because these are cytoplasmic proteins. The concentration of all other proteins is plotted per area unit because they are cell envelope bound. The cell cycle age is plotted as percentage of the mass doubling time (80 min) of the to steady state grown MC4100 cells.

The Map of Profiles can be used to determine the amount of fluorescence present in an individual cell. From the morphological parameters, the volume or the surface area of each cell can be calculated and therefore the relative concentration of the immunolabeled protein can be determined. We analyzed the cellular concentration of many morphogenetic proteins as function of the division cycle (**Figure 4**) and noticed that most proteins have a constant concentration at all cell ages (**Table 3**). The genes coding for a number of the cytoplasmic steps of PG precursor synthesis and several of the cell division proteins such as *ftsL*, *ftsI* (*pbpB*), *ftsW*, *ftsQ*, *ftsA,* and *ftsZ* are expressed from one large operon called the *dcw* cluster (Vicente et al., 1998). Other genes such as *ftsN* and *ftsK* are in separated locations on the chromosome. Multiple promoters regulate the expression of *ftsZ*. The *ftsQ* p1gearbox promoter was reported to ensure the cell size dependent constant concentration of FtsQ, FtsA, and FtsZ under various growth conditions (Aldea et al., 1990; Sitnikov et al., 1996; Ballesteros et al., 1998). The ratio between the number of proteins produced per cell cycle in cells grown in rich medium and cells grown in poor medium is fairly constant for many of the morphogenetic proteins (Li et al., 2014). Therefore, these σ^S^ dependent promoter types might also be involved in the expression of other morphogenetic proteins. However, as far as we are aware not much is known about the promoter organization of their genes. Even less is known about the regulation of gene expression as a function of the cell cycle, which is clearly a gap in our present knowledge.

**Table 3:** Immunolabeled proteins.

Protein	Function	Change in concentration (%)^a^	Number of cells	Anti-serum purification	Concentration based various techniques (source)^b^	Concentration based on (Li et al., 2014)
FtsZ	Z-ring	25	3528	Serum is specific	4800 ± 1300 (Mohammadi et al., 2009)	3335
FtsA	Membrane tether of FtsZ and divisome protein recruitment	n.d.	n.d.	n.d.	200 (Mukherjee and Donachie, 1990)	575
ZipA	Membrane tether of FtsZ and FtsA modulator.	10	6555	ZipA depleted		501
ZapA	Cross-links Z, binds ZapB	18	8378	ΔZapA	6100 ± 1000 (Mohammadi et al., 2009)	738
ZapB	Binds ZapA and MatP	20	5050	ΔZapB	~13000 (Ebersbach et al., 2008)	7797
FtsK	Divisome activation and chromosome deconcatenation	10	6505	Affinity	~100 (Bisicchia et al., 2013b)	213
FtsB	Binds FtsQ	10	5110	Affinity		140
PBP3	Transpeptidase	40	5499	Affinity	63 ± 12 (Dougherty et al., 1996)	144
FtsN	Divisome activator	40	5921	Serum is specific	4650 ± 1780 ref (Ursinus et al., 2004)	269
PBP1B	Glycosyl transferase and transpeptidase	10	3522	ΔPBP1B	123 ± 19 (Dougherty et al., 1996)1000 (Paradis-Bleau et al., 2010)	139
PBP1A	Glycosyl transferase and transpeptidase	10	3138	Affinity andΔPBP1A	135 ± 24 (Dougherty et al., 1996) 500 (Paradis-Bleau et al., 2010)	116
LpoB	PBP1B activator	10	5177	ΔLpoB	2300 (Paradis-Bleau et al., 2010)	954
LpoA	PBP1A activator	10	5670	ΔLpoA	500 (Paradis-Bleau et al., 2010)	250
PBP5	DD-carboxypeptidase	30	5997	ΔPBP5	317 ± 69 (Dougherty et al., 1996)	1180
MinC^c^	FtsZ inhibitor	15	6110	ΔMinCDE	400 ± 80 (Szeto et al., 2001)	148
MinD^c^	MinC tethering to membrane	10	4315	ΔMinCDE	3000 (de Boer et al., 1991) 2000 (Shih et al., 2002)	644

^a^An increase of 10% is to be expected for all samples as the controls eosine for cytoplasmic proteins and bodipy for membrane proteins, which are expected to have a constant concentration, also increase 10% in concentration during the cell division cycle. Therefore, the effective change in concentration will be for all proteins the given value-10%. ^b^The numbers are derived from a variety of different strains an growth conditions and therefore not directly comparable to the numbers in the last column. ^c^See Supplementary Figure S1 for the concentration of MinD, MinC and eosine as a function of the cell cycle age.

The constant cellular concentration of the immunolabeled proteins indicated that despite their transition from single, dimeric or subcomplex protein state to their presence in a multi-protein complex during the assembly of the division machinery, epitopes remained accessible to the antibodies. Antibody epitopes are usually directed at exposed and flexible regions of the protein, especially when the antibodies are developed against purified protein as it is the case for our sera. The proteins that had a variable concentration during the cell cycle will be discussed individually below.

When the number of proteins per average cell is known, it is possible to translate the fluorescent units of the immunolabeling into number of proteins. Knowing the number of proteins and their localization in the cell envelope gives the possibility to determine the number of proteins in the divisome or the stoichiometry of its subunits. The resolution of the microscope is not enough to restrict the localization measurements to the precise position of the septal ring. Therefore we used a much larger volume of midcell extended by 0.4 μm on either side of the center. Conceding that not all proteins in this volume are part of the septal ring, we calculated the number of molecules that were present in this volume above the general background of the same molecules in the cell (MolsCPlus). We did not choose the alternative method to simply include all molecules in the central volume because this most certainly would have resulted in an overestimation. To allow comparison, both sets of data are provided in the Supplementary Table S1.

Based on immunoblotting the average number of FtsZ molecules per cell was calculated to be 4800 ± 1300 (*n* = 3) in our wild type strain MC4100 grown to steady state in Gb1 at 28°C with a mass doubling time of 80 min (average cell volume is 1.35 ± 0.27 μm^3^; Mohammadi et al., 2009). These are exactly the same conditions that have been used for the growth of the same strain in the present paper (see Materials and Methods). For MG1655 cells grown in MOPS minimal medium with a mass doubling time of 56 min (average cell volume is 1.43 ± 0.31 μm^3^), the average number of FtsZ molecules was determined by ribosome profiling to be 3335 ± 1300 molecules (Li et al., 2014). The difference in average cell volume between these two strains and growth conditions is negligible given the 30% error (Li et al., 2014) in the determination of the number of proteins per average cell. This method seems to be relatively accurate and it has been performed for all proteins on a single strain grown under well-defined conditions. Therefore, we used the mean number of molecules per average cell determined by ribosome profiling (Li et al., 2014) for all calculations on the number of absolute molecules at midcell. Other data on the mean number of molecules found in the literature are often based on less reliable methods such as immunoblotting and are obtained from a large variety of growth conditions and strains (for comparison, the various measurements are presented in **Table 3**). Although Li et al. (2014) have taken the life-time of the proteins into account for their calculation of the number of proteins per cell, they could not correct for regulated protein degradations such as ClpX degradation of FtsZ (Camberg et al., 2009) or for fractions of proteins that are not active. Therefore, the absolute numbers presented here can be subject to variation.

The calculation of the volume or surface of the cells is based on the phase contrast images. To avoid over-interpretation of the results, we have also labeled the membrane with a fluorophore (bodipy-C12) and the cytosol with a fluorescent dye (eosine) and determined their cellular concentration as function of the cell cycle in fluorescence units. Both display an increase of 10% during the cell cycle (shown for bodipy in **Figure 4** and for eosine in Supplementary Figure S1). Consequently, only an increase of a protein concentration measured during the cell cycle of more than 10% was considered relevant. In addition, we argued that the concentration of a protein at the end of the cell cycle should be close to or decreasing toward the concentration of the protein in new-born cells. Changes in concentration that did not abide to this rule were not considered significant.

### The Proto-Ring

**FIGURE 5:**
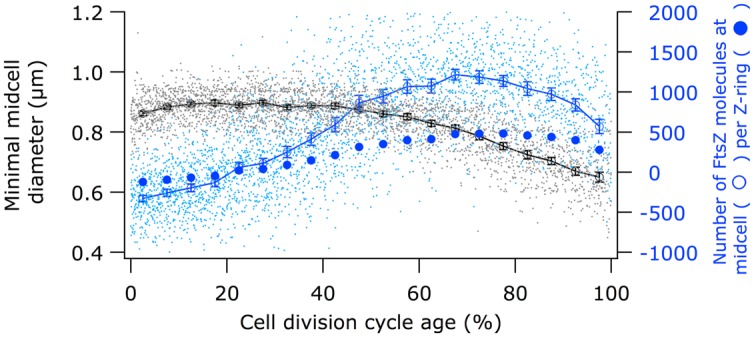
**Comparison of the timing of the constriction with the timing of the FtsZ localization at midcell.** Black circles (legend on the left) show minimal cell diameter (constriction) versus cell age. Open blue circles (legend on the right) show “MolsCPlus,” which is the number of extra FtsZ molecules at midcell compared to the number of FtsZ in the cell assuming an equal distribution of the molecules in the cytosol. Filled blue circles (legend on the right) show the mean number of FtsZ molecules per μm Z-ring. The bottom axis shows the cell division cycle age in percentage.

Using immunolabeling and the Coli-Inspector macro, an increase of 15% in the cellular concentration of FtsZ was observed (**Figure 4**). Interestingly, 13% of the FtsZ molecules are degraded per generation (Camberg et al., 2009). Possibly, the number of molecules per cell is regulated by the two component protease ClpX that is known to be involved in the degradation (Camberg et al., 2011, 2014). The average number of 3335 FtsZ molecules per cell (Li et al., 2014) was used to calculate the concentration of FtsZ proteins at midcell. Between 60 and 80% of the cell age about 1100 ± 77 FtsZ molecules are present at midcell within 95% confidence borders (Supplementary Table S1). This is in agreement with the reported present of 30% of the total number of FtsZ molecules present at midcell (Stricker et al., 2002). Based on total internal reflection (TIR) PALM imaging, the Z-ring could consist of loosely organized protofilaments of limited size (Fu et al., 2010; Buss et al., 2013). The width of the Z-ring (110 nm) is largely invariant between different bacterial species and FtsZ expression levels (Fu et al., 2010; Jennings et al., 2011; Biteen et al., 2012). The length of the average protofilament in the ring could be about 120 nm or contain 27 FtsZ subunits (Stricker et al., 2002; Anderson et al., 2004; Chen and Erickson, 2005; Loose and Mitchison, 2014). With 1100 FtsZ molecules at midcell the Z-ring would consist of about 40 of these protofilaments. The number of FtsZ molecules/μm Z-ring at midcell increased up to 60% of the cell cycle age and thereafter remained constant at ∼450 molecules/ μm till 90% of the cell cycle age (**Figure 5**; Supplementary Figure S1). The calculation of the circumference of the cell is based on the measured minimal midcell diameter (**Figure 5**) and the assumption that the three envelope layers constrict simultaneously. The resolution of the images was not good enough to use the minimal midcell diameter after 90% of the cell cycle for further calculations of the progression of the closure of the constriction. Summarizing, it can be concluded that the density of the Z-ring increases during the initial constriction and then stays constant.

ZipA and FtsA anchor FtsZ protofilaments to the cytoplasmic membrane (Ma et al., 1996; Mosyak et al., 2000; Yan et al., 2000; Hale and de Boer, 2002; Kuchibhatla et al., 2011). ZipA was recently shown to protect FtsZ against degradation by the ClpXP protease (Pazos et al., 2013a). ZipA is also thought to prevent self-interaction of FtsA because ZipA is not essential in an FtsA mutant that is not able to self-interact (Pichoff et al., 2012). The ZipA cellular concentration is constant during the cell division cycle (**Figure 4**) and ∼170 ± 15 molecules are observed at midcell (Supplementary Table S1). Assuming the FtsA cellular concentration also to be constant (Rueda et al., 2003) and knowing that 30% of the FtsA molecules is present in the Z-ring (Pla et al., 1990), about 200 FtsA molecules would bind to the Z-ring. Consequently, each protofilament of 27 FtsZ residues would therefore be bound to the cell envelope by 4 ZipA molecules and 5 FtsA molecules. ZipA has a high affinity for FtsZ and interacts with the flexible C-terminus of FtsZ of which especially residue D373 is essential for the interaction (Haney et al., 2001). As FtsA binds with a lower affinity to the same flexible C-terminal domain of FtsZ (amino acids 367–383; Szwedziak et al., 2012), they will likely not bind simultaneously to the same FtsZ molecule. ZipA can form dimers (Skoog and Daley, 2012) and could therefore bind as dimer two FtsZ molecules in a single FtsZ protofilament but it could also bind two FtsZ protofilaments given its long and flexible cytoplasmic domain. In conclusion, together FtsA and ZipA could link every third FtsZ molecule to the cytoplasmic membrane.

The cellular concentration of ZapB was constant during the cell cycle (**Figure 4**). The cellular concentration of ZapA increased up to 30% cell age, stayed constant up to 70% cell age after which it decreased again to the level of new-born cells. The number of ZapA molecules at midcell did not mimic this change in concentration. ZapA is present at midcell with 150 ± 10 -170 ± 12 molecules between 67.5 and 92.5% of the cell age, indicating that while the total cellular ZapA concentration is already decreasing, the number of molecules at midcell is still increasing. The majority of the ZapA molecules is present as a tetramer in *E. coli* (Low et al., 2004; Small et al., 2007; Mohammadi et al., 2009; Pacheco-Gómez et al., 2013), which implies that every FtsZ protofilament can be cross-linked by a ZapA tetramer assuming that the ZapA tetramer can at least bind two protofilaments. ZapA does not bind the flexible C-terminal domain of FtsZ but binds to the core domain (den Blaauwen, unpublished results) allowing for mutual binding of ZapA and ZipA or FtsA. ZapB localized at midcell with 4200 ± 250 molecules between 72.5 and 92.5% of the cell cycle. ZapB is a dimer and is dependent on its binding to ZapA (Galli and Gerdes, 2010) and possibly also on FtsZ (Pazos et al., 2013b) for its localization at midcell. Clearly, not enough ZapA molecules are present to bind all ZapB molecules [even if assuming the number of midcell ZapA molecules to be 1400 as determined by immunoblotting (Mohammadi et al., 2009)]. Galli and Gerdes (2010) could discriminate a fluorescent ZapB ring localizing inside the Z-ring by confocal microscopy. Given the axial resolution of the microscope a distance of about 200 nm would be required to resolve both structures. This is not compatible with a single ZapA tetramer connecting FtsZ and ZapB (**Figure 7**). ZapB is able to bind MatP, a protein that binds to specific sequences abundant in the terminal region of the chromosome (Mercier et al., 2008; Espeli et al., 2012). It would make sense if the ZapB molecules would extend toward the chromosome during the constriction process to verify whether the chromosomes are sufficiently separated. Recently, a high resolution microscopy study was published that confirms the presence of a layered network of FtsZ-ZapA-ZapB-MatP molecules (Buss et al., 2015). Interaction of ZapB with the chromosome might be communicated to the Z-ring and somehow stall the progress of the constriction to avoid cleavage of the nucleoids.

### The Septal Synthesizing Complex

#### Protein Concentrations of PBP3 and FtsN Fluctuate

PBP3 is a transpeptidase that crosslinks peptides in peptidoglycan specifically during cell division at midcell (Adam et al., 1997; Weiss et al., 1999; Piette et al., 2004). PBP3 forms a subcomplex (Fraipont et al., 2011) with FtsW, which is possibly one of the peptidoglycan precursor lipid-II flippases (Ruiz, 2008; Mohammadi et al., 2011, 2014; Sham et al., 2014). PBP3 is essential for septal peptidoglycan synthesis, and its inhibition by aztreonam or its depletion results in a division arrest (Pogliano et al., 1997; Eberhardt et al., 2003). PBP3 interacts with PBP1B (Banzhaf et al., 2012), a bifunctional peptidoglycan synthesizing protein with glycosyl transferase activity to polymerize glycan strands and transpeptidase activity. The activity of PBP1B is stimulated by its interactions with LpoB (Paradis-Bleau et al., 2010; Typas et al., 2010) and FtsN (Müller et al., 2007). The cellular concentration of PBP1B and LpoB was constant during the cell division cycle of *E. coli* (**Figure 4**). Similarly, the bifunctional PBP1A involved in cell elongation as well as its regulator LpoA had a constant cellular concentration during the division cycle (**Figure 4**). Remarkably, the cellular concentration of PBP3 molecules increased as soon as it started to accumulate at midcell at 40% of the cell age until it reached a maximum at about 70% after which it returned to its level before midcell localization. The cellular concentration of PBP3 increased by 30% during it localization at midcell. At its maximum cellular concentration about 70 ± 6 PBP3 molecules were present at midcell. Assuming it to be a dimer (Fraipont et al., 2011; Sauvage et al., 2014), 35 ± 3 peptidoglycan synthesizing proteins complexes could be present in the Z-ring or approximately one per average FtsZ protofilament (see for images of the immunolocalization Supplementary Figure S2). Curiously, only maximally 18 ± 3 molecules of PBP1B were present at midcell above the cellular background, which is not sufficient to interact with each PBP3 dimer. The number of possible interactions is further reduced if PBP1B exists as a dimer (Zijderveld et al., 1991; Bertsche et al., 2005). Surprisingly, dimers of PBP1B or PBP1A, or a PBP1B-PBP1A complex were not observed using our in cell FRET assay with the fluorescent labeled proteins (Alexeeva et al., 2010; see Supplementary information and Table S2 and S3). The absence of FRET does not proof that PBP1B or 1A are monomers. However, evidence that the bifunctional PBPs are dimers *in vivo* is thus far lacking. The imbalance in the number of PBP molecules is not resolved by assuming that all molecules in the center cell volume are part of the divisome (Supplementary Table S1). Although PBP1B and PBP1A have been shown to be involved in cell division and cell elongation, respectively (Bertsche et al., 2006; Banzhaf et al., 2012), they can substitute for each other (Typas et al., 2010). Moreover, the elongasome and divisome have been reported to interact at least temporarily during septal synthesis (Vats et al., 2009; Fenton and Gerdes, 2013; van der Ploeg et al., 2013) and therefore, the 7 ± 2 PBP1A molecules present in surplus to the background of PBP1A molecules at midcell should be added to the septal peptidoglycan synthesizing complexes. The resulting 25 bifunctional PBP molecules (or 12.5 dimers) in the divisome are still not sufficient to saturate the 35 PBP3 dimers. Because of the multitude of interactions of PBP1B with other cell division proteins, occlusion of the epitopes of the polyclonal IgG might reduce the number of detectable PBP1B molecules at midcell resulting in an underestimation of protein numbers. However, if a subset of PBP1B molecules would become inaccessible because of association with divisome proteins one would expect the concentration of PBP1B to decrease in dividing cells unless the number of PBP1B molecules is upregulated during cell division like observed for FtsZ.

Two other proteins that are thought to be part of the core complex of the synthetic complex, FtsK and FtsB, were immunolabeled. FtsK has two functions, its integral membrane domain is needed to recruit the FtsQLB complex (Wang and Lutkenhaus, 1998), and it is involved in the coupling of the simultaneous constriction of the cytoplasmic membrane and the peptidoglycan layer (Berezuk et al., 2014). The cytoplasmic domain of FtsK is needed for its second function to position the *dif* sites near the terminus of the chromosomes to allow the XerCD recombinases (Löwe et al., 2008) to decatenate the chromosomes. The cytoplasmic domain consists of a long flexible linker and a DNA translocating γ-domain, which forms at least during ds-DNA translocation a double hexamer (Massey et al., 2006). Using a chromosomally encoded FtsK-YPet FP fusion between 24 and 36 FtsK molecules were observed at midcell during the constriction period in minimal medium grown cells (Bisicchia et al., 2013b). Because the measured number was mostly a multiplication of six, it was concluded that FtsK forms a hexamer at the site of constriction. In agreement with the data of (Bisicchia et al., 2013b), we observe between 50 ± 8 and 60 ± 8 FtsK molecules at midcell (Supplementary Table S1), which would be sufficient for 8–10 hexamers.

FtsB is part of the FtsQLB complex (Buddelmeijer and Beckwith, 2004; van den Berg van Saparoea et al., 2013) that interacts with many of the divisome proteins (Karimova et al., 2005, 2009; D’Ulisse et al., 2007). The interaction of this complex with FtsN was recently shown to activate cell division (Liu et al., 2015). FtsQ, FtsB, and FtsL are present with 147, 140, and 201 molecules per average cell, respectively (Li et al., 2014) and form a complex with a 1:1:1 stoichiometry (Luirink and den Blaauwen, unpublished results). Based on the immunolocalization of FtsB, about 20 ± 3 of these complexes will localize at midcell. Using the same method as for FtsK, Bisicchia et al. (2013b) detected between 36 and 66 FtsQ molecules at midcell in constricting cells using a chromosomally encode YPet-FtsQ FP fusion. Based on the periodicity of the numbers it was concluded that FtsQ, like FtsK, occurred as a hexamer. In our experience it is very difficult to obtain antibodies against the individual FtsQ, FtsL, FtsB, and FtsK proteins that give good and specific signal in cells (den Blaauwen and Luirink, unpublished results). Therefore, we cannot exclude that some of the FtsB epitopes are not accessible in the FtsQBL complex and that not all FtsB molecules present at midcell are detected. Based on our data and the data of (Bisicchia et al., 2013b) between 3 and 11 hexameric FtsQBL complexes could be present in the divisome. In view of a limited number of synthetic complexes, the observed 18 PBP1B plus 7 PBP1A molecules at midcell might not be an underestimation.

Taken our data and those of Bisicchia et al. (2013b) together 36–60 FtsK, 20–66 FtsQ, 25 PBP1A/B, and 70 PBP3 molecules could be present at midcell during constriction. If a hexameric configuration of FtsK is assumed, about 6–10 septal synthesizing complexes could be envisioned. The presence of 3–4 bifunctional peptidoglycan synthases per synthetic complex would allow the simultaneous insertion of 3–4 glycan strands. Such a mode of peptidoglycan synthesis would fit with the Höltje (1998) hypothesized model in which one glycan strand of the existing peptidoglycan layer is replaced by three new glycan strand or the “three for one model.” The uncertainty in the number of proteins at midcell could easily be explained by the error in the measurement of the mean number of proteins in the cell (Li et al., 2014) and the error in the immunolocalization given the very low protein copy-numbers. A detailed PALM/STORM analysis of the stoichiometry of the divisome protein might be able to provide conclusive numbers.

The essential FtsN protein is a bitopic membrane protein with a short cytoplasmic domain that interacts with the IC domain of FtsA (Busiek and Margolin, 2014). Followed in the periplasm by an extended region that begins with three short helices of which the second is essential (Yang et al., 2004; Gerding et al., 2009). This region ends with a C-terminal SPOR domain that interacts with peptidoglycan (Ursinus et al., 2004) that may only be transiently present during the division process (Gerding et al., 2009). FtsN binds with its amino terminal 6 amino acids the IC domain of FtsA and keeps FtsA in a monomeric state that is able to recruit the peptidoglycan synthetic complex (Pichoff et al., 2014). The essential helix of FtsN is likely to affect the conformation of the FtsQBL complex because mutants in FtsL and FtsB can bypass FtsN (Liu et al., 2015). The SPOR domain (Duncan et al., 2013) is essential for self-enhanced localization at midcell of FtsN (Gerding et al., 2009; Rico et al., 2013). FtsN overexpression allows the bypass of ZipA because it fixes FtsA in the monomeric state by interacting with its IC domain. Overproduction of FtsN, but also the presence of the FtsL and FtsB mutants that can bypass FtsN, results in very short cells that initiate septal synthesis at a much earlier stage (Pichoff et al., 2014; Liu et al., 2015; Tsang and Bernhardt, 2015). This is possibly caused by a much faster recruitment of the late cell division proteins than in the wild type situation. By interacting with FtsA, by affecting FtsQLB, and by interacting with the peptidoglycan synthetic complex as well as with peptidoglycan, FtsN is likely able to monitor and secure the synchrony of the envelope synthesis.

The cellular concentration of FtsN is more or less constant up to 40% of the cell age before it starts to increase until it reaches a maximum at 90% of the cell age (**Figure 4** and see for images of the immunolocalization Supplementary Figure S2). The number of FtsN molecules at midcell also increased continuously until a maximum of about 150 ± 8 molecules is reached at 90% cell age (Supplementary Table S1). Consequently, the density of the number of FtsN molecules/μm Z-ring continues to increase at midcell at a cell age where the maximum number of FtsZ and PBP3 molecules is already declining, which agrees with its reported self enhanced localization during constriction (Gerding et al., 2009) and its self interaction (Di Lallo et al., 2003; Karimova et al., 2005; Alexeeva et al., 2010). The increase in cellular protein concentration for FtsN is, like for PBP3, ∼30% (**Figure 4**; **Table 2**).

That the number of FtsN molecules decreases much later than the number of FtsZ and PBP3 molecules at midcell is in agreement with the model of (Pichoff et al., 2014) in which the reduction of the number of FtsZ molecules at midcell leaves less FtsZ molecules available for the weakly FtsZ binding FtsA in competition with ZipA. The higher density of FtsN molecules will ensure that sufficient monomeric FtsA will be available to successfully compete with ZipA for the reduced number of FtsZ molecules.

#### The PBP5 Concentration Varies with Cell Age

PBP5 is the major DD-carboxypeptidase during exponential growth in *E. coli*. It Localizes at midcell in a substrate dependent fashion (Potluri et al., 2010). PBP5 started to accumulate at midcell at about 35% of the cell age, continued to accumulate and reached a maximum at 80% with 550 ± 32 molecules after which it decreased (Supplementary Table S1). This accumulation at midcell is partly due to a 20% increase in the PBP5 concentration during constriction (**Figure 4**). With its abundance at midcell, most pentapeptides that are not immediately used for the formation of peptide cross-links during septal peptidoglycan synthesis by PBP3/PBP1B and/or PBP1A/PBP2 will be converted to tetrapeptides by PBP5.

**FIGURE 6:**
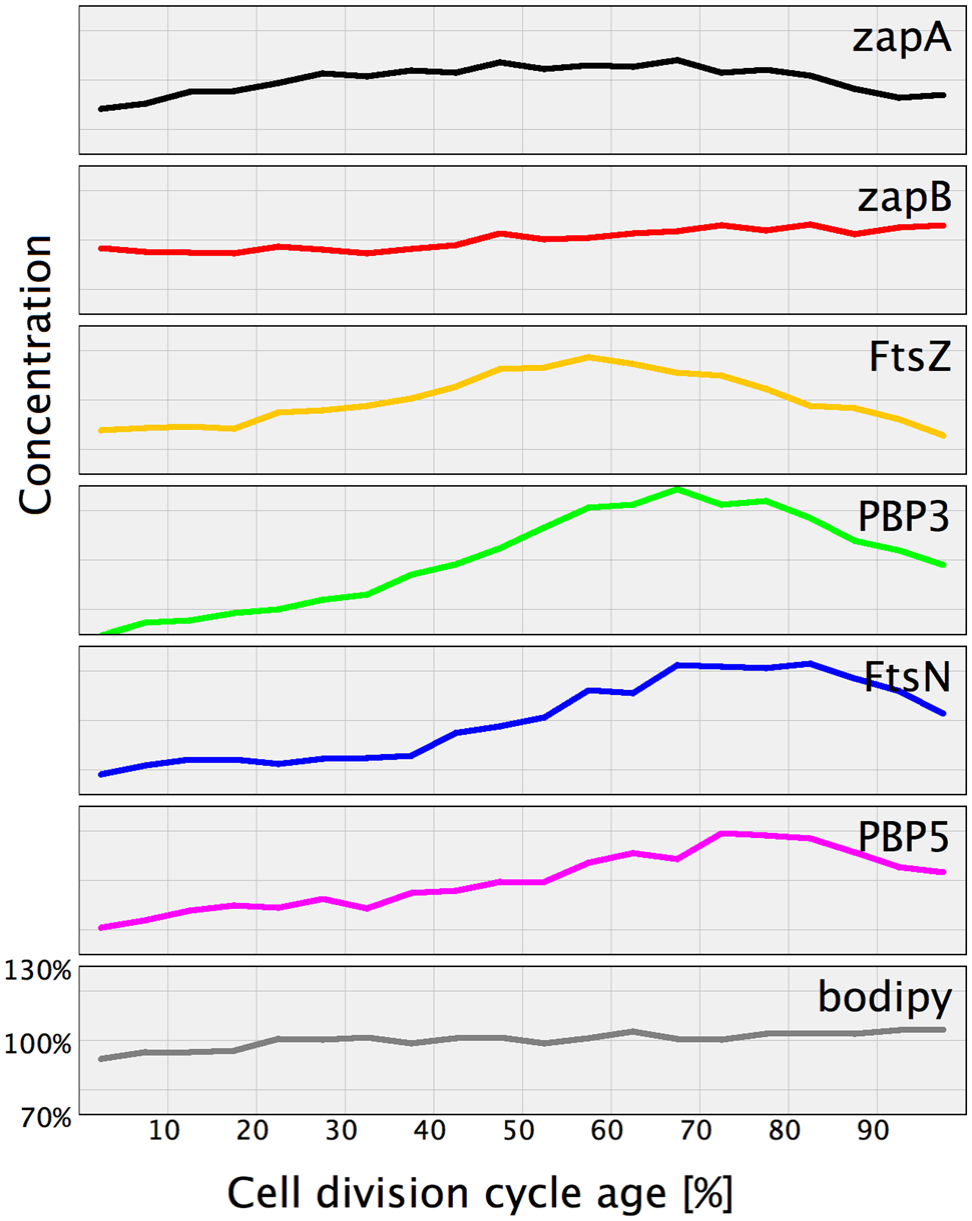
**Normalized protein concentration as function of cell age.** For comparison the proteins that were found to have a cell age dependent concentration variation have been plotted in one graph. The concentration at the various cell cycle time points of each indicated protein was divided by the average concentration of that protein in the whole population. Subsequently, the concentration for the individual proteins was plotted against the cell division cycle age in percentage with an off sett on the *Y*-axes to enable individual visualization. For comparison the membrane stain bodipy-C12 and the ZapB protein that both have a constant concentration are included.

Of the immunolocalized proteins, FtsZ, PBP3, FtsN, and PBP5 increased their cellular concentration during constriction (**Figure 6**). Consequently, the excess of these proteins has to be removed by either proteolytic degradation or by regulation of their expression. It has been suggested by Camberg et al. (2011) that ClpXP may be involved in division by degrading other proteins than FtsZ. Maybe it also degrades PBP3 and FtsN, but not PBP5 which is periplasmic.

## Conclusion

**FIGURE 7:**
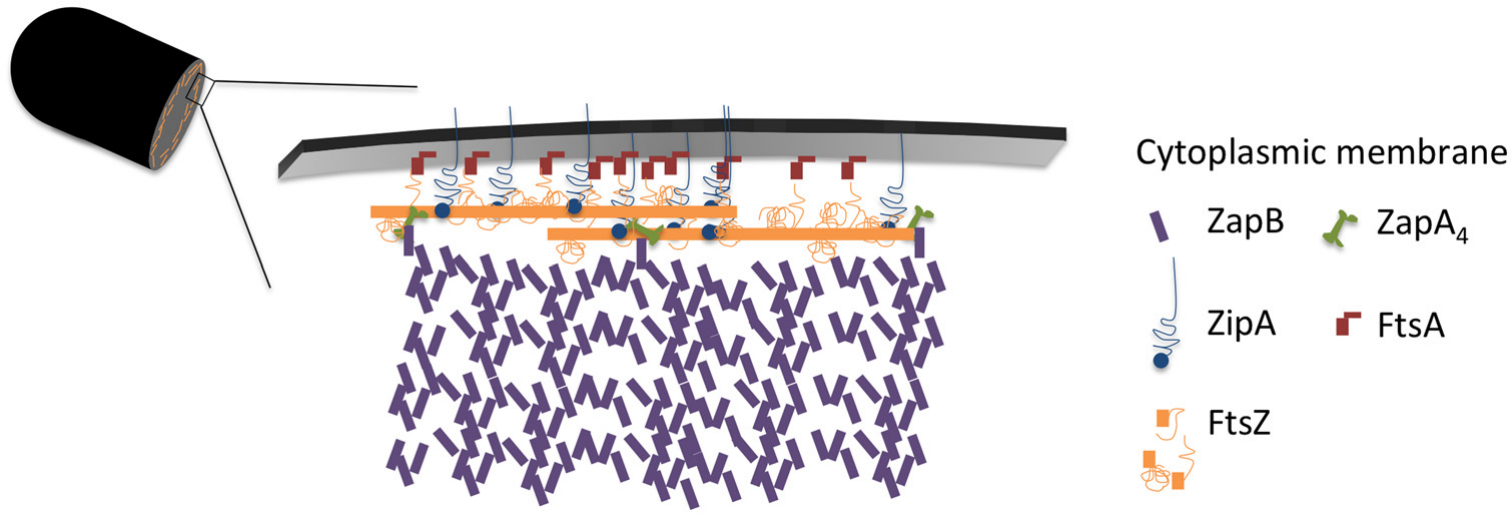
**Model of the proto-ring.** Two FtsZ filaments in orange, each consisting of 27 residues with a length of 120 nm are connected to the cytoplasmic membrane by 4 ZipA molecules (blue) or 2 ZipA dimers that might cross-link 2 FtsZ protofilaments and 5 FtsA molecules (red). ZipA and FtsA compete for binding to the flexible C-terminal end of FtsZ. Each FtsZ protofilament is bound to a ZapA tetramer (green) that is potentially able to cross-link the protofilaments and which is also bound by ZapB (purple). About 100 ZapB molecules are available for each protofilament.

Immunolocalization analyzed as function of cell age allowed determination of the concentration of the labeled proteins and revealed that at least the concentration of FtsZ, ZapA, PBP3, FtsN, and PBP5 seem to be cell cycle regulated (**Figure 6**). Using the published mean number of proteins per cell, it was also possible to establish a stoichiometry for the proto-ring. For every protofilament of ∼27 FtsZ residues, 4 ZipA, 5 FtsA, 1 ZapA_4_, and 105 ZapB molecules are available (**Figure 7**). Every second to fourth protofilament could also contain one peptidoglycan synthetic complex of which the composition might vary. When FtsN is included, it could bind 4 out of the 5 FtsA molecules that are present on an FtsZ protofilament. While cell division is in progress and the septum is closing, the number most divisome proteins, except FtsZ and PBP3, seem to be constant up to ∼90% cell age. This indicates that the molecule density of the divisome increases and that the amount of new envelope added to the closing septum is constant as was suggested in Wientjes and Nanninga (1989).

## Conflict of Interest Statement

The authors declare that the research was conducted in the absence of any commercial or financial relationships that could be construed as a potential conflict of interest.

## Footnotes

1 https://sils.fnwi.uva.nl/bcb/objectj/

2 https://www.micro-manager.org

3 http://imagej.nih.gov/ij/

4 https://sils.fnwi.uva.nl/bcb/objectj/

5 https://sils.fnwi.uva.nl/bcb/objectj/examples/AlignFluorChannels/AlignFluor
