# Supplementary material for: Cell age dependent concentration of Escherichia coli divisome proteins analyzed with ImageJ and ObjectJ
Source: Front Microbiol. 2015 Jun 11;6:586. doi: 10.3389/fmicb.2015.00586 (PMC4462998; doi:10.3389/fmicb.2015.00586)
Supplement: Supplementary file 1 [file Data_Sheet_1.PDF]

MolsCPlus/age (%)	FtsN	95%conf FtsN	ZipA	95%conf ZipA	ZapB	95%conf ZapB	PBP3	95%conf PBP3	ZapA	95%conf ZapA	PBP1A	95%conf PBP1A	PBP1B	95%conf PBP1B	LpoB	95% conf LpoB	LpoA	95% conf LpoA	PBP5	95% conf PBP5	FtsZ	95%conf FtsZ	FtsK	95%conf FtsK	FtsB	95%conf FtsB
3	-39	3	-74	7	-1157	127	-6	2	-71	7	-7	2	-13	2	-74	14	-17	3	-67	10	-329	39	-33	4	-9	3
8	-31	3	-58	7	-725	162	-3	2	-62	7	-5	2	-12	2	-45	14	-16	3	-59	9	-259	46	-27	4	-7	3
13	-28	3	-59	7	-484	177	-3	2	-61	8	-7	3	-10	3	-37	13	-13	3	-49	11	-195	50	-29	4	-4	2
18	-27	3	-43	7	-23	195	-1	2	-52	9	-5	2	-7	2	-40	13	-9	3	-24	9	-129	54	-26	4	-2	3
23	-18	3	-37	7	421	216	0	2	-26	9	-5	2	-9	2	-27	14	-10	3	-20	10	60	62	-25	4	-2	3
28	-11	3	-23	7	821	215	2	2	-6	10	-6	2	-7	3	-26	13	-10	3	-7	12	109	64	-17	4	1	3
33	-7	4	-11	7	1341	216	7	3	11	9	-3	2	-8	2	-26	14	-4	3	15	13	252	70	-23	4	1	3
38	4	5	-6	7	1968	194	13	3	29	10	-4	2	-7	2	-6	14	-5	3	48	15	412	76	-13	4	1	3
43	15	5	3	8	2289	182	22	4	62	10	-1	3	-2	2	-5	13	-3	3	77	16	593	88	-11	4	3	3
48	36	7	23	8	2771	208	33	4	86	11	-3	2	1	3	-1	14	0	3	151	20	865	94	-1	6	1	3
53	53	7	43	8	2982	174	41	5	98	10	-1	2	4	3	15	15	0	3	207	22	948	85	4	4	4	3
58	81	8	52	9	3243	194	54	6	119	10	-2	2	5	3	32	16	4	4	282	25	1069	84	9	6	5	3
63	90	8	60	11	3478	153	60	6	134	11	1	2	5	3	49	17	7	3	373	26	1075	86	22	6	8	3
68	114	8	84	11	3882	190	72	6	151	10	2	2	10	3	62	19	12	3	398	27	1217	65	23	6	6	3
73	132	7	112	13	4181	209	65	5	152	10	4	2	12	3	90	18	18	4	490	30	1187	78	34	7	8	3
78	143	8	128	12	4139	214	70	5	168	11	7	3	15	3	116	23	27	4	529	31	1138	68	48	7	15	3
83	156	8	142	14	4459	258	64	6	170	12	7	2	18	3	134	23	26	4	553	32	1043	85	54	8	18	3
88	158	8	168	15	4203	257	57	6	155	13	8	2	20	3	133	21	38	4	522	32	973	78	56	8	22	3
93	149	9	153	13	4160	353	52	6	152	13	10	3	20	3	147	24	38	4	492	35	840	77	60	8	23	3
98	119	10	144	16	2729	500	41	6	116	13	11	3	20	3	135	22	36	5	427	35	571	89	46	8	25	4

Minimal midcell diameter	Minimal midcell circumference
0.86	2.71
0.88	2.77
0.89	2.80
0.90	2.82
0.89	2.79
0.90	2.82
0.88	2.77
0.89	2.79
0.89	2.79
0.87	2.75
0.86	2.70
0.85	2.67
0.83	2.60
0.81	2.55
0.79	2.47
0.75	2.37
0.72	2.28
0.70	2.21
0.67	2.10
0.65	2.04

MolsCPlus is the number of molecules present at midcell in surplus to the rest of the cell

Number of molecules are derived from the number of molecules per average cell published in Li, G.-W., Burkhardt, D., Gross, C., & Weissman, J. S. (2014). Quantifying absolute protein synthesis rates reveals principles underlying allocation of cellular resources. Cell, 157(3), 624–635. doi:10.1016/j.cell.2014.02.033

VolCenterMols/age (%)	FtsN	95%conf FtsN	ZipA	95%conf ZipA	ZapB	95%conf ZapB	PBP3	95%conf PBP3	ZapA	95%conf ZapA	PBP1A	95%conf PBP1A	PBP1B	95%conf PBP1B	LpoB	95% conf LpoB	LpoA	95% conf LpoA	PBP5	95% conf PBP5	FtsZ	95%conf FtsZ	FtsK	95%conf FtsK	FtsB	95%conf FtsB
3	74	3	170	7	2225	87	39	2	240	8	44	2	47	3	278	13	94	3	374	12	1034	48	69	3	51	3
8	80	3	170	6	2534	108	44	2	254	7	44	3	50	3	291	16	99	4	376	11	1088	47	72	4	50	3
13	82	3	168	6	2703	128	45	2	259	8	47	3	55	3	313	15	98	3	419	13	1181	57	70	3	49	3
18	87	4	182	7	2895	143	47	2	286	8	41	2	51	3	301	14	103	4	419	14	1139	57	69	3	48	3
23	83	4	177	6	3299	162	49	2	291	11	48	3	51	3	315	15	99	4	432	16	1387	74	74	4	53	3
28	96	4	188	7	3424	161	52	2	333	11	45	3	56	3	306	15	106	4	433	15	1351	69	76	4	50	4
33	91	5	200	7	3727	179	55	3	314	10	49	3	57	3	302	15	101	3	439	17	1518	78	72	4	50	3
38	103	6	197	8	4135	186	64	3	348	12	47	3	57	3	321	16	110	4	470	18	1572	84	77	4	49	3
43	111	6	204	7	4333	181	70	4	353	11	52	3	54	3	316	15	101	4	494	17	1826	102	75	4	47	3
48	132	8	215	8	4811	214	81	4	380	11	46	3	58	3	318	15	111	4	547	22	1999	105	87	6	46	3
53	139	7	230	10	4792	175	89	5	370	11	47	3	57	3	323	18	101	4	586	24	2088	99	85	5	47	3
58	171	8	228	10	5000	212	101	6	378	11	46	3	59	4	333	17	105	5	661	27	2157	98	97	6	48	3
63	171	8	241	12	5165	161	106	6	381	11	47	3	52	3	333	20	98	4	713	27	2114	103	100	6	48	4
68	197	8	252	12	5423	195	116	6	385	12	41	3	58	4	335	19	102	4	730	27	2182	87	99	7	44	3
73	210	7	272	14	5651	227	108	5	366	11	43	3	54	4	348	20	108	5	817	30	2079	86	108	8	48	3
78	212	9	279	13	5547	234	111	6	375	12	44	3	56	4	358	25	106	5	831	34	1945	94	115	8	50	4
83	223	9	282	14	5825	255	103	7	359	14	41	3	55	4	371	23	103	5	848	35	1816	106	121	8	53	4
88	219	9	301	16	5496	270	95	6	335	12	40	3	57	4	361	22	113	5	791	36	1709	92	118	9	55	4
93	207	9	282	14	5535	355	87	6	324	14	43	3	55	3	357	26	106	5	739	38	1522	88	116	9	53	4
98	177	11	271	16	4384	433	76	6	296	13	42	3	52	4	331	25	107	6	672	40	1256	91	104	8	58	4

VolCenterMols is the number of molecules present at the cell center ± 0.4 µm

Number of molecules are derived from the number of molecules per average cell published in Li, G.-W., Burkhardt, D., Gross, C., & Weissman, J. S. (2014). Quantifying absolute protein synthesis rates reveals principles underlying allocation of cellular resources. Cell, 157(3), 624–635. doi:10.1016/j.cell.2014.02.033

Moleculea*µm-1/age (%)	FtsN	95%conf FtsN	ZipA	95%conf ZipA	ZapB	95%conf ZapB	PBP3	95%conf PBP3	ZapA	95%conf ZapA	PBP1A	95%conf PBP1A	PBP1B	95%conf PBP1B	LpoB	95% conf LpoB	LpoA	95% conf LpoA	PBP5	95% conf PBP5	FtsZ	95%conf FtsZ	FtsK	95%conf FtsK	FtsB	95%conf FtsB
3	-14	1	-27	3	-427	47	-2	1	-26	3	-2	1	-5	1	-27	5	-6	1	-25	4	-121	14	-12	2	-3	1
8	-11	1	-21	2	-261	58	-1	1	-22	3	-2	1	-4	1	-16	5	-6	1	-21	3	-94	17	-10	1	-3	1
13	-10	1	-21	2	-173	63	-1	1	-22	3	-3	1	-4	1	-13	5	-5	1	-17	4	-70	18	-10	1	-2	1
18	-10	1	-15	3	-8	69	0	1	-19	3	-2	1	-2	1	-14	5	-3	1	-8	3	-46	19	-9	1	-1	1
23	-6	1	-13	2	151	77	0	1	-9	3	-2	1	-3	1	-10	5	-4	1	-7	3	22	22	-9	2	-1	1
28	-4	1	-8	2	291	76	1	1	-2	4	-2	1	-3	1	-9	5	-3	1	-2	4	39	23	-6	2	0	1
33	-3	1	-4	2	484	78	2	1	4	3	-1	1	-3	1	-9	5	-2	1	5	5	91	25	-8	2	1	1
38	1	2	-2	3	705	69	5	1	11	4	-1	1	-2	1	-2	5	-2	1	17	5	148	27	-5	2	0	1
43	5	2	1	3	822	65	8	1	22	4	-1	1	-1	1	-2	5	-1	1	27	6	213	32	-4	2	1	1
48	13	2	8	3	1009	76	12	2	31	4	-1	1	0	1	0	5	0	1	55	7	315	34	0	2	0	1
53	20	2	16	3	1103	64	15	2	36	4	0	1	1	1	5	6	0	1	76	8	351	31	1	2	2	1
58	30	3	19	3	1214	73	20	2	45	4	-1	1	2	1	12	6	1	1	106	9	400	32	3	2	2	1
63	34	3	23	4	1336	59	23	2	52	4	0	1	2	1	19	6	3	1	143	10	413	33	8	2	3	1
68	45	3	33	4	1520	74	28	2	59	4	1	1	4	1	24	8	5	1	156	11	476	26	9	2	2	1
73	54	3	45	5	1693	84	26	2	61	4	1	1	5	1	36	7	7	2	199	12	481	31	14	3	3	1
78	60	4	54	5	1750	91	30	2	71	5	3	1	6	1	49	10	11	2	224	13	481	29	20	3	6	1
83	68	4	62	6	1960	113	28	3	75	5	3	1	8	1	59	10	12	2	243	14	458	37	24	3	8	2
88	72	4	76	7	1903	116	26	3	70	6	4	1	9	1	60	9	17	2	236	14	440	35	26	4	10	1
93	71	4	73	6	1977	168	25	3	72	6	5	1	10	1	70	12	18	2	234	17	399	37	28	4	11	2
98	58	5	70	8	1337	245	20	3	57	7	6	1	10	1	66	11	18	2	209	17	279	44	22	4	12	2

The MolsCPlus divided by the curcumfernce at midcell using the minimal midcell diameter plotted in figure 5.
