# Supplementary material for: Cell age dependent concentration of Escherichia coli divisome proteins analyzed with ImageJ and ObjectJ
Source: Front Microbiol. 2015 Jun 11;6:586. doi: 10.3389/fmicb.2015.00586 (PMC4462998; doi:10.3389/fmicb.2015.00586)
Supplement: Supplementary file 2 [file Data_Sheet_2.PDF]

Supplementary information

Stoichiometry and cell age dependent concentration of *Escherichia coli* divisome proteins

Norbert O.E. Vischer[1*], Jolanda Verheul[1], Marten Postma[1], Bart van den Berg van Saparoea[1,3], Kenn Gerdes[5], Elisa Galli[2], Joen Luirink[3], Waldemar Vollmer[2], Miguel Vicente[4] Paolo Natale[4,6] and Tanneke den Blaauwen[1*]

Table S1. Excell file containing the mean FCPlusMol and mean RingFraction data in 5% age bins of morphogenetic proteins. Each datapoint is the mean value of a 5% age bin and the column on its right contains the corresponding values of the 95% confidence interval. The first column contains the age classes.

Supplementary introduction

Are PBP1B and PBP1A dimers? On a native SDS-PAGE PBP1B runs as dimer and as monomer (Zijderveld et al., 1991). Isolated PBP1B molecules associate on a surface plasmon resonance chip with a K_D of 12 µM (Bertsche et al., 2005). Given the concentration of ~ 1.8 µM PBP1B in the envelope, not all PBP1Bs are expected to form dimers. PBP1B and 1A are bitopic membrane proteins with cytoplasmic domains of 64 and 5 amino acids, respectively. Their glycosyltransferase and transpeptidase enzymatic domains are in the periplasm. PBP1B is observed as α, β, and γ versions (Tamaki et al., 1977) that all have GTase as well as transpeptidase activities (Nakagawa and Matsuhashi, 1982). The α-version contains the full-length protein of 844 amino acids, whereas the γ–version starts at methionine 46 (Broome-Smith et al., 1985; Kato et al., 1984). The β component is a degradation product of the α-version and lacks the first 24 amino acids (Suzuki et al., 1987). All three forms of PBP1B run on a SDS-Page as monomers and as dimers. The cytoplasmic domain is not required for dimerization, but seems to be involved in correct folding of PBP1B when it is present (Chalut et al., 2001). PBP1A (850 amino acids) was also reported to be a dimer independent from PBP1B (Charpentier et al., 2002). To determine whether PBP1B and PBP1A could be observed to form dimers in the cells, we used our FRET assay (Alexeeva et al., 2010; Fraipont et al., 2011).

Supplementary materials and methods

E. coli strains LMC500 (MC4100*lysA; F, araD139,Δ(argF-lac)U169, deoC1, flbB5301, ptsF25, rbsR, relA1, rpslL150, lysA1*) (Taschner et al., 1988), JW3359 (BW25113*mrcA; F-,*

rrnB3, ΔlacZ4787, hsdR514, Δ(araBAD)567, Δ(rhaBAD)568, rph-1, mrcA::kan) and JW0145 (BW25113*mrcB; F-, rrnB3, ΔlacZ4787, hsdR514, Δ(araBAD)567, Δ(rhaBAD)568, rph-1, mrcB::kan*) (Baba et al., 2006) were grown as described by Alexeeva et al. (Alexeeva et al., 2010). These host strains contained combinations of plasmids for expression of cell division proteins PBP1A, PBP1Bα or PBP1Bγ fused to fluorescent proteins mKO or mCherry (Table S3). The plasmids expressing the various fusion proteins were transform to strain EJ801 *F^-, lac, strA, tonA, metB, proA, tsx, ponA1104, ponB1085, dacA1191, dacB12* (Hara and Suzuki, 1984). The cells were grown in TY (10g of bactotryptone, 5g of yeast extract, 5 g of NaCl, 15 mmol NaOH per liter) at 28°C to mid exponential phase and than shifted to the non-permissive temperature of 42°C after which continuation of normal growth was confirmed for the strain expressing the fusion proteins but not for the strain that contained the plasmid only. Standard PCR and cloning techniques were used for DNA manipulation.

Supplementary results and discussion

No PBP1 dimers observed in cells using FRET. Fluorescent protein fusions were made to the α- as wel as to the γ-versions of PBP1B and to PBP1A and their functionality was tested. All FP-fusion were able to complement the growth defect of strain EJ801, which harbors the Δ*mcrB*/*mrcA*(ts) genes, at the non-permissive temperature (not shown), indicating that they are fully functional. Subsequently, their interactions were probed with FRET. In this assay, cells grown in minimal medium at 28°C were fixed as described (Alexeeva et al., 2010) and donor and acceptor spectra of the MKO and mCherry fluorophores were measured in the fixed cells in a cuvette using a fluorescence spectrophotometer. The FRET efficiency was determined by unmixing the emission spectrum of the donor excited FRET pair into a donor component, the direct excitation of the acceptor, the background, and the FRET signal (see for a detailed description of the method (Alexeeva et al., 2010; van der Ploeg et al., 2013). No interactions were observed (Table S2), whereas the Ef_A, (apparent efficiency of energy transfer of the acceptor) of the positive control, a direct fusion of the donor and the acceptor fluorescent proteins, was in all experiments about 0.30.

The orientation of the donor and acceptor fluorescent proteins of the protein fusions can be such that the fluorophores are farther apart than ~9 nm and will not resonate. This can be caused by the presence of bridging proteins that prevent a direct interaction but also because of an unusual orientation of the fluorophores. A final possibility is that PBP1A and PBP1B are dimerizing while they are synthesized, which would result in dimers that contain always either two mKO fusion proteins or two mCherry proteins that will not FRET. Measurements of the dimerization of many of the other PBPs present in *E. coli* yielded FRET efficiencies of about 5-10% (T. den Blaauwen unpublished results) showing that absence of FRET is not a characteristic of PBPs in general. Our conclusion is therefore that our failure to measure the dimerization of PBP1B and PBP1A might indicate that they do not function as dimers in the cell. Additional experiments are needed to obtain conclusive data on the dimeric state of the bifunctional PBP1s in the cell.

Table S2 FRET efficiencies Ef_A are shown for combinations of cell division proteins PBP1A, PBP1Bα and PBP1Bγ in different *E. coli* hosts.

**Host**	**Protein combination**	*****	**Donor**	**Acceptor**	**FRET efficiency Ef_A #**
LMC500	PBP1A + PBP1A	Ac	mKO-PBP1A	mCh	0.012
		Dc	mKO	mCh-PBP1A	0.016
		FP	mKO-PBP1A	mCh-PBP1A	0.010
JW3359 (Δ*mrcA*)	PBP1A + PBP1A	Ac	mKO-PBP1A	mCh	0.016
		Dc	mKO	mCh-PBP1A	0.018
		FP	mKO-PBP1A	mCh-PBP1A	0.036
LMC500	PBP1Bα + PBP1Bα	Ac	mKO-PBP1Bα	mCh	0.025
		Dc	mKO	mCh-PBP1Bα	-0.005
		FP	mKO-PBP1Bα	mCh-PBP1Bα	-0.001
JW0145 (Δ*mrcB*)	PBP1Bα + PBP1Bα	Ac	mKO-PBP1Bα	mCh	-0.012
		Dc	mKO	mCh-PBP1Bα	-0.006
		FP	mKO-PBP1Bα	mCh-PBP1Bα	0.006
LMC500	PBP1Bγ + PBP1Bγ	Ac	mKO-PBP1Bγ	mCh	0.014
		Dc	mKO	mCh-PBP1Bγ	-0.005
		FP	mKO-PBP1Bγ	mCh-PBP1Bγ	0.020
LMC500	PBP1Bα + PBP1Bγ	Ac	mKO-PBP1Bα	mCh	-0.001
		Dc	mKO	mCh-PBP1Bγ	-0.005
		FP	mKO-PBP1Bα	mCh-PBP1Bγ	0.030
LMC500	PBP1Bγ + PBP1Bα	Ac	mKO-PBP1Bγ	mCh	0.014
		Dc	mKO	mCh-PBP1Bα	-0.005
		FP	mKO-PBP1Bγ	mCh-PBP1Bα	0.020
JW3359 (Δ*mrcA*)	PBP1A + PBP1Bγ	Ac	mKO-PBP1A	mCh	0.016
		Dc	mKO	mCh-PBP1Bγ	ND
		FP	mKO-PBP1A	mCh-PBP1Bγ	0.015
JW3359 (Δ*mrcA*)	PBP1Bγ + PBP1A	Ac	mKO-PBP1Bγ	mCh	ND
		Dc	mKO	mCh-PBP1A	0.018
		FP	mKO-PBP1Bγ	mCh-PBP1A	0.030

* Ac, acceptor control; Dc, donor control; FP, fusion pair

# Ef_A, apparent efficiency of energy transfer of the acceptor; ND not determined.

Table S3. Plasmids for expression of mKO or mCherry and for expression of cell division proteins PBP1A, PBP1Bα or PBP1Bγ fused to fluorescent proteins mKO or mCherry.

**Plasmid name**	**Protein to be expressed**[2]	**Donor vector or PCR template/primers**	**Recipient vector**	**Digestion**[1]	**Ref.**
pSAV058	mKO	-	-	-	(Alexeeva et al., 2010)
pSAV047	mCherry	-	-	-	(Alexeeva et al., 2010)
pBB003	mCherry-PBP1A	PCR pWA001/5'-atagaattcaacaacaacgtgaagttcgtaaagtat-3' and 5'-atcaccgccatttcgttc-3'	pWA001 (mCherry-PBP1A expression plasmid)	E/X	This work
pBB004	mKO-PBP1A	pBB003	pSAV058	E/H	This work
pBB008[b]	mCherry-PBP1Bα (M46A)	pGFP-PBP1Bα (M46A)	pBB012 (mCherry fusion cloning plasmid)	E/B	This work
pBB009	mCherry-PBP1Bγ	pGFP-PBP1Bγ	pBB012	E/B	This work
pBB010	mKO-PBP1Bα (M46A)	pGFP-PBP1Bα (M46A) and pWA002	pSAV057	E/B and N/E	(Alexeeva et al., 2010) and this work
pBB011	mKO-PBP1Bγ	pGFP-PBP1Bγ and pWA001	pSAV057	E/B and N/E	(Alexeeva et al., 2010) and this work

[1]E, *EcoR*I; X, *Xho*I; H, *Hind*III; B, *BamH*I; N, *Nco*I. [2]Linker between mCherry or mKO and PBP1B or PBP1A consist of the amino acids; mKO-PBP1A…AVAHS**EFNNN**VKFVK…, mKO-PBP1Bα…AVAHS**EFNNN**AGNDR…, mKO-PBP1Bγ …AVAHS**EFNNN**APRKG…, mCherry-PBP1A…DELYK**EFNNN**VKFVK…, mCherry-PBP1Bα…DELYK**EFNNN**AGNDR…, mCherry-PBP1Bγ…DELYK**EFNNN**APRKG…

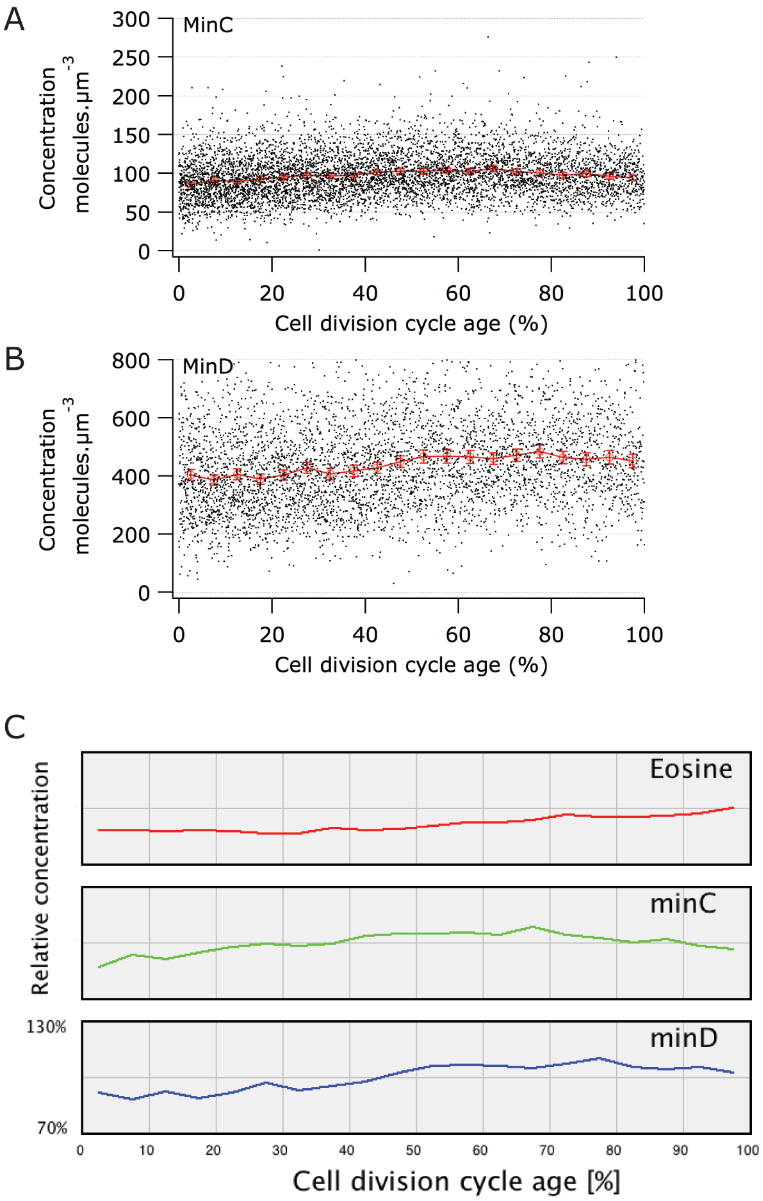

Figure S1. Concentration of the MinC and MinD proteins as function of cell division cycle age. For each graph the concentration of the indicated protein is plotted against the cell age in %. The black dots are the data for each individual cell. The red line and markers are the mean value of 5% age bins and the error bars indicate the border of the 95% confidence interval. The concentration of MinC (A) and MinD **(B)** is plotted as volume unit because these are cytoplasmic proteins. The cell cycle age is plotted as percentage of the mass doubling time (80 min) of the to steady state grown MC4100 cells. Summary of the timing of the concentration fluctuations of MinC and MinD (C). In comparison, the cytoplasmic stain eosine has a constant concentration. Plot frames represent 70% .. 130% of the mean concentration (vertical) and 0%..100% cell age (horizontal).

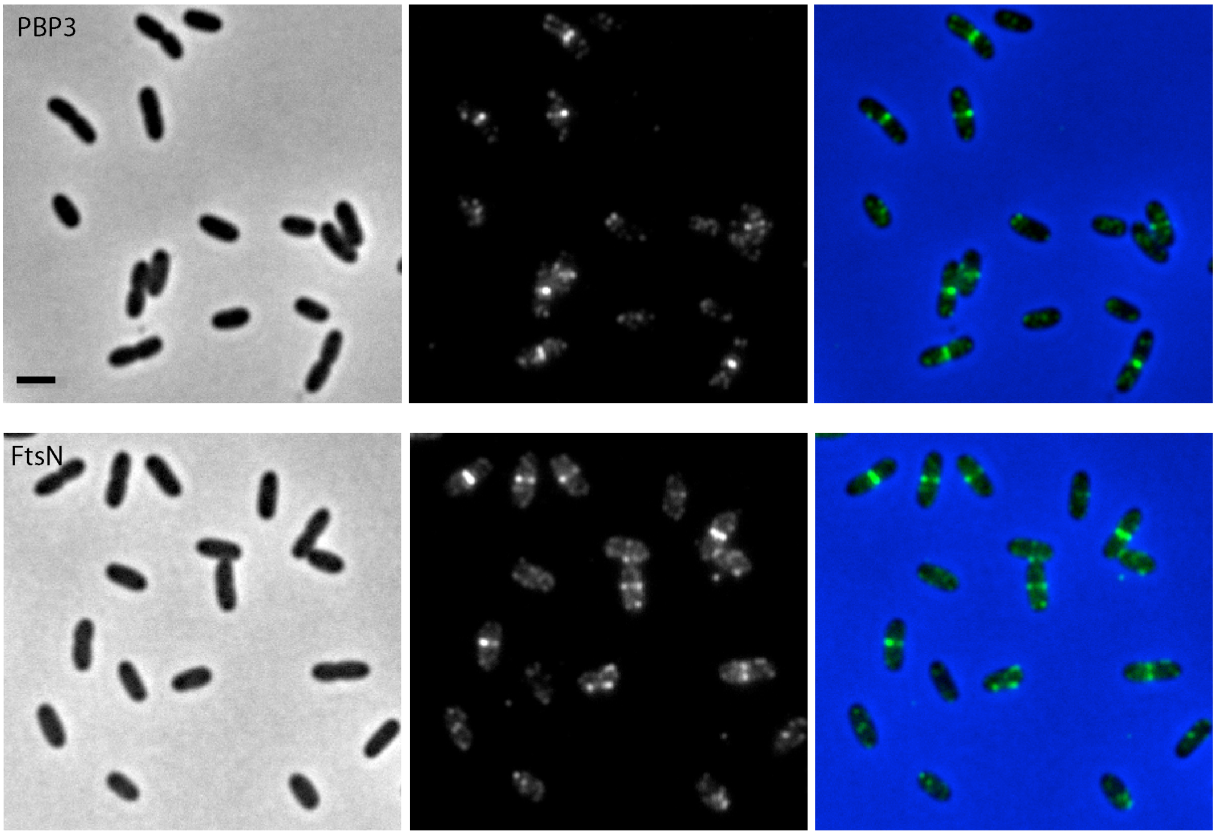

Figure S2. Immunolocalization of PBP3 and FtsN. Phase contrast, fluorescence and a merge image of both are shown from left to right for PBP3 (upper row) and for FtsN (lower row). The scale bar equals 2 μm.

Supplementary references

Alexeeva, S., Gadella, T. W. J., Verheul, J., Verhoeven, G. S., and Blaauwen, den, T. (2010). Direct interactions of early and late assembling division proteins in *Escherichia coli* cells resolved by FRET. *Mol Microbiol* 77, 384–398. doi:10.1111/j.1365-2958.2010.07211.x.

Baba, T., Ara, T., Hasegawa, M., Takai, Y., Okumura, Y., Baba, M., Datsenko, K. A., Tomita, M., Wanner, B. L., and Mori, H. (2006). Construction of *Escherichia coli* K-12 in-frame, single-gene knockout mutants: the Keio collection. *Mol Syst Biol* 2, 2006.0008. doi:10.1038/msb4100050.

Bertsche, U., Breukink, E., Kast, T., and Vollmer, W. (2005). In vitro murein peptidoglycan synthesis by dimers of the bifunctional transglycosylase-transpeptidase PBP1B from *Escherichia coli*. *J Biol Chem* 280, 38096–38101. doi:10.1074/jbc.M508646200.

Broome-Smith, J. K., Edelman, A., Yousif, S., and Spratt, B. G. (1985). The nucleotide sequences of the *pon*A and *pon*B genes encoding penicillin-binding protein 1A and 1B of Escherichia coli K12. *Eur J Biochem* 147, 437–446.

Chalut, C., Charpentier, X., Remy, M. H., and Masson, J. M. (2001). Differential responses of Escherichia coli cells expressing cytoplasmic domain mutants of penicillin-binding

protein 1b after impairment of penicillin-binding proteins 1a and 3. *J Bacteriol* 183, 200–206. doi:10.1128/JB.183.1.200-206.2001.

Charpentier, X., Chalut, C., Rémy, M.-H., and Masson, J.-M. (2002). Penicillin-binding proteins 1a and 1b form independent dimers in *Escherichia coli*. *J Bacteriol* 184, 3749–3752. doi:10.1128/JB.184.13.3749-3752.2002.

Fraipont, C., Alexeeva, S., Wolf, B., van der Ploeg, R., Schloesser, M., Blaauwen, den, T., and Nguyen-Distèche, M. (2011). The integral membrane FtsW protein and peptidoglycan synthase PBP3 form a subcomplex in Escherichia coli. *Microbiology* 157, 251–259. doi:10.1099/mic.0.040071-0.

Hara, H., and Suzuki, H. (1984). A novel glycan polymerase that synthesizes uncross-linked peptidoglycan in Escherichia coli. *FEBS Lett* 168, 155–160.

Kato, J.-I., Suzuki, H., and Hirota, Y. (1984). Overlapping of the coding regions for α and γ components of penicillin-binding protein 1 b in *Escherichia coli. Molec. Gen. Genet.* 196, 449–457. doi:10.1007/BF00436192.

Nakagawa, J., and Matsuhashi, M. (1982). Molecular divergence of a major peptidoglycan synthetase with transglycosylase-transpeptidase activities in *Escherichia coli* --- penicillin-binding protein 1Bs. *Biochem Biophys Res Commun* 105, 1546–1553.

Suzuki, H., Kato, J., Sakagami, Y., Mori, M., Suzuki, A., and Hirota, Y. (1987). Conversion of the alpha component of penicillin-binding protein 1b to the beta component in Escherichia coli. *J Bacteriol* 169, 891–893.

Tamaki, S., Nakajima, S., and Matsuhashi, M. (1977). Thermosensitive mutation in Escherichia coli simultaneously causing defects in penicillin-binding protein-1Bs and in enzyme activity for peptidoglycan synthesis in vitro. *Proc Natl Acad Sci USA* 74, 5472–5476.

Taschner, P. E., Huls, P. G., Pas, E., and Woldringh, C. L. (1988). Division behavior and shape changes in isogenic *ftsZ, ftsQ, ftsA, pbpB*, and *ftsE* cell division mutants of Escherichia coli during temperature shift experiments. *J Bacteriol* 170, 1533–1540.

van der Ploeg, R., Verheul, J., Vischer, N. O. E., Alexeeva, S., Hoogendoorn, E., Postma, M., Banzhaf, M., Vollmer, W., and Blaauwen, den, T. (2013). Colocalization and interaction between elongasome and divisome during a preparative cell division phase in Escherichia coli. *Mol Microbiol* 87, 1074–1087. doi:10.1111/mmi.12150.

Zijderveld, C. A., Aarsman, M. E., Blaauwen, den, T., and Nanninga, N. (1991). Penicillin-binding protein 1B of *Escherichia coli* exists in dimeric forms. *J Bacteriol* 173, 5740–5746.
